# Individualized lesion-symptom mapping using explainable artificial intelligence for the cognitive impact of white matter hyperintensities

**DOI:** 10.1016/j.nicl.2025.103790

**Published:** 2025-04-18

**Authors:** Ryanne Offenberg, Alberto De Luca, Geert Jan Biessels, Frederik Barkhof, Wiesje M. van der Flier, Argonde C. van Harten, Ewoud van der Lelij, Josien Pluim, Hugo Kuijf

**Affiliations:** a Image Sciences Institute, UMC Utrecht, Utrecht, the Netherlands; b Department of Neurology, UMC Utrecht, Utrecht, the Netherlands; c Department of Radiology & Nuclear Medicine, Amsterdam UMC, Vrije Universiteit, the Netherlands; d Queen Square Institute of Neurology and Centre for Medical Image Computing, University College London, UK; e Alzheimer Center Amsterdam, Neurology, Vrije Universiteit Amsterdam, Amsterdam UMC Location VUmc, Amsterdam, the Netherlands; f Amsterdam Neuroscience, Neurodegeneration, Amsterdam, the Netherlands; g Epidemiology & Data Science, Vrije Universiteit Amsterdam, Amsterdam UMC Location VUmc, Amsterdam, the Netherlands; h Department of Biomedical Engineering, Eindhoven University of Technology, Eindhoven, the Netherlands

**Keywords:** Lesion-symptom mapping, Neural network, Explainable artificial intelligence, Vascular cognitive impairment, Machine learning

## Abstract

Lesion-symptom mapping methods assess the relationship between lesions caused by cerebral small vessel disease and cognition, but current technology like support vector regression (SVR)) primarily provide group-level results. We propose a novel lesion-symptom mapping approach that can indicate how lesion patterns contribute to cognitive impairment on an individual level. A convolutional neural network (CNN) predicts cognitive scores and is combined with explainable artificial intelligence (XAI) to map the relation between cognition and vascular lesions.

This method was evaluated primarily using real white matter hyperintensity maps of 821 memory clinic patients and simulated cognitive data, with weighted lesions and noise levels. Simulated data provided ground truth locations to assess predictive performance of the CNN and accuracy of strategic lesion identification by XAI, using an established lesion-symptom mapping method, SVR, and a simple fully connected neural network (FNN) as benchmarks. Real cognitive scores were used in a final proof-of-principle analysis.

Predictive performance in simulation experiments was high for the CNN (R^2^ = 0.964), SVR (R^2^ = 0.875), and FNN (R^2^ = 0.863). CNN with XAI provided patient-specific attribution maps that highlighted the ground truth locations. All methods showed similar sensitivity to noise. Using real cognitive scores, SVR (R^2^ = 0.291) obtained a somewhat higher predictive performance than the CNN (R^2^ = 0.216), although both methods substantially exceeded the predictive performance of total WMH volume alone (R^2^ = 0.013). The FNN performed worse on real data (R^2^ = 0.020).

To conclude, results show that CNNs combined with XAI can perform lesion-symptom mapping and generate individual attribution maps, which could be a valuable feature with further method development.

## Highlights

• First step towards novel lesion-symptom mapping method.
• Combination of a neural network and eXplainable AI.
• Patient-specific maps that indicate lesion location importance.
• Novel method achieves comparable performance to benchmarks.
• Application on simulated and real cognitive data.

## Graphical abstract

**Figure:**
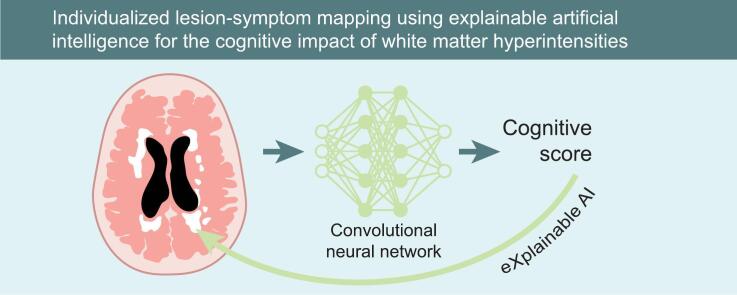

## 1 Introduction

Vascular lesions in the brain can cause cognitive impairment, affecting different cognitive domains such as attention and executive functioning, but also memory (Weaver et al., 2021, Hamilton et al., 2021). For brain infarcts (Weaver et al., 2021, Zhao et al., 2018), and also for white matter hyperintensities (WMH) (Coenen et al., 2023), lesion location influences cognitive impact. In literature, such lesion locations with particular cognitive impact are referred to as “strategic”. Lesion-symptom mapping (LSM) is increasingly used to identify strategic lesion locations by examining the relationship between lesion locations in the brain and a behavioural score in large datasets on a group level (Bates et al., 2003, de Haan and Karnath, 2018). This is done by statistically associating lesioned voxels in binary lesion maps (created by segmenting lesions of interest from brain scans) and behavioural scores, such as cognitive tests scores.

A variety of LSM methods have been developed, from mass-univariate voxel-based (Weaver et al., 2021, Coenen et al., 2023, Bates et al., 2003, Rorden et al., 2007) to multivariate analyses (Zhang et al., 2014, Pustina et al., 2018, Sperber et al., 2019, Zhao et al., 2018). In mass-univariate methods, statistical testing is applied to every voxel in the lesion map to evaluate if the presence of a lesion is associated with a difference in cognitive scores. Because each voxel is evaluated independently, this method cannot take interrelations between lesion locations into account. Multivariate methods, such as support vector regression (SVR), analyse a lesion pattern as a whole, retaining relations between voxels. This is relevant, because vascular lesions occur with distinct patterns depending on underlying mechanisms and are therefore not randomly distributed throughout the brain. Next to that, different lesion locations can have combined or interacting effects on cognition, because the brain is organized in (functional) networks. A downside of both mass-univariate and multivariate approaches is that they provide results on a group level and cannot readily indicate how lesion patterns contribute to an individual patient’s cognitive impairment, while this could guide impactful use-cases for personalized diagnosis and treatment.

The use of neural networks might be a promising novel approach for LSM. A neural network, when offered sufficient training data, should be able to learn the relationship between vascular lesions and cognitive scores, enabling the model to predict cognitive scores for every patient. Neural networks are inherently flexible models that can handle multiple inputs and outputs, which would be of value for LSM. Convolutional neural networks (CNNs) are commonly used to extract information from medical images (Lee et al., 2017, Sarvamangala and Kulkarni, 2022). For instance, CNNs have been used previously to predict brain age (Jonsson, et al., 2019, Lee et al., 2022) and a multitude of brain diseases, including dementia (Khan et al., 2021). Moreover, Chaucan et al. (2019) employed a CNN to predict cognitive scores based on stroke lesion maps (Chauhan et al., 2019). The integration of explainable artificial intelligence (XAI) methods could extend the use of a CNN from prediction model to LSM method. XAI could enable the identification of strategic lesion locations, in addition to making the neural network interpretable. A key difference from conventional LSM methods, is the potential to support use-cases that need to identify strategic lesion patterns at the level of an individual patient.

In this work we introduce and evaluate the use of a CNN combined with XAI to perform LSM on WMH. SVR and a simple fully connected neural network (FNN) were used as benchmarks. Evaluating LSM methods is challenging because cognitive performance is influenced by factors beyond a lesion image. By definition, LSM methods can only partially explain cognitive variance, and without ground truth strategic lesion locations, methods can only be compared head-to-head for evaluation. Moreover, identified lesion locations can only be evaluated against prior knowledge. We addressed these limitations by generating simulated cognitive data using actual lesion maps. This provided a ground truth to which both predictive performance for cognition and accuracy of strategic lesion identification were assessed. Our principal experiments were therefore performed using simulated cognitive data. Additionally, different lesion weights and effects of noise were evaluated. Finally, we tested the methods using WMH maps and real attention and executive functioning cognitive scores from a large memory clinic cohort, to show a proof-of-principle application of combining a CNN and XAI in individual patients.

## 2 Materials & methods

### 2.1 Study design

A CNN was trained to predict (simulated) cognitive scores of memory clinic patients based on the segmentation mask of their WMH. During training, the model captured the relationship between WMH and (simulated) cognitive score. Subsequently, XAI was used to determine the strategic lesion locations for each patient individually. This was done by computing the attribution of all WMH locations to the predicted score for each individual patient’s lesion map. Two methods were used as benchmark for our proposed LSM approach: SVR, a conventional LSM method that could be considered as gold standard, and a FNN. Three controlled experiments to validate our approach were performed on simulated cognitive data: (i) a basic simulation of cognitive data, (ii) with modified regional weights, and (iii) with added noise. Furthermore, the LSM methods were applied on the real attention and executive functioning scores.

### 2.3 Lesion-symptom mapping approaches

#### 2.3.1 Convolutional neural network

**Fig. 1:**
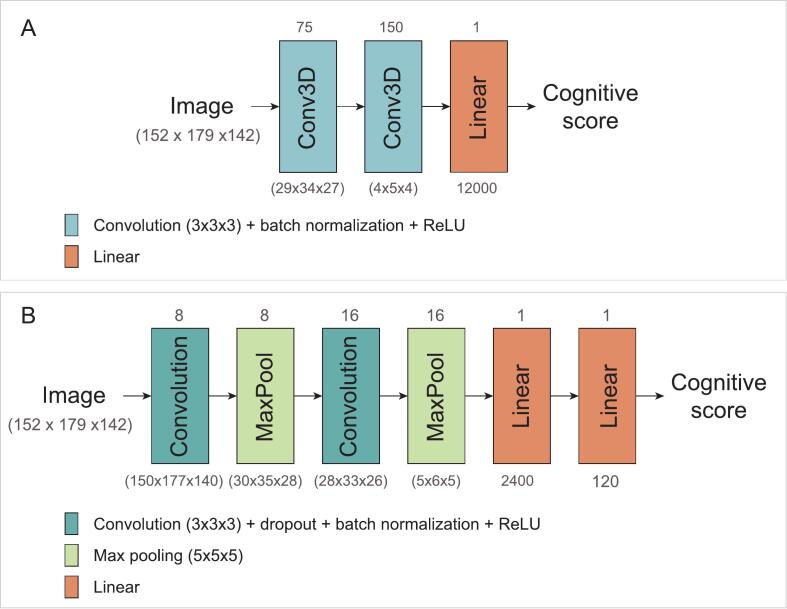
**CNN architecture overview.** The CNN architecture used in the simulation experiments (A) and the architecture used in the experiments with the real attention and executive functioning scores (B).

CNNs contain convolutional layers that summarize the information of the input image into a smaller representation using a sliding convolution kernel. A simple 3D CNN consisting of two convolutional blocks was used in the simulation study, see Fig. 1A. Since the real cognitive data is far more complex, this network was adapted to include more regularization for the experiment using the real attention and executive functioning scores. Specifically, a dropout layer was included in the convolution block, a max pooling layer was added after each convolution block, and a second linear layer was included (Fig. 1B). The analyses with simulated data did not require the more regularized model.

The number of considered voxels (i.e. features) was reduced in the experiment using the real attention and executive functioning scores to reduce input dimensionality, as was done in the benchmark methods, through feature selection. Relevant voxels were identified by mass-univariate LSM and nulled in the 3D WMH maps (Zhao et al., 2018, Kuijf, 2021). We implemented a standard mass-univariate approach as described previously (Zhao et al., 2018). For the simpler experiments on simulated data, the full 3D WMH maps were used as input for the CNN model. Volume correction was not applied, a neural network is able to learn such a feature if necessary.

Post hoc XAI, specifically a method called occlusion, was employed to obtain the attribution maps (Zeiler and Fergus, 2014, Kokhlikyan et al., 2020). Occlusion is a perturbation-based method and works by analysing the change in prediction as a result of occluding parts of the lesion image using a sliding window. Occluding locations with high attribution result in bigger changes in prediction compared to locations with little attribution. Iteratively, the importance of each image location was computed and summarized in an attribution map, see Fig. 4 for some examples (Borys et al., 2023). A 5 × 5 × 5 voxels sliding window was used in combination with a 3 × 3 × 3 voxels stride. Only the positive attribution values were used in the analyses.

#### 2.3.2 Support vector regression

SVR with a radial basis function kernel was implemented. Its parameters were optimized in a 5-fold cross-validation grid search using scikit’s GridSearchCV function (Pedregosa et al., 2011). Performance with and without feature selection was also compared. The combination of parameters resulting in the highest Pearson correlation between predicted and true values in the test fold was chosen.

WMH maps were corrected for total lesion volume using an established approach, which involves dividing lesioned voxels by the square root of the total lesion volume (Zhang et al., 2014). Subsequently, feature selection was applied by removing any voxels deemed irrelevant by mass-univariate LSM. Finally, images were flattened into a one-dimensional array. SVR beta-maps were used as attribution maps.

#### 2.3.3 Fully connected neural network

In addition to the CNN and SVR models, a simple fully connected neural network was used. The reason to include this model was to have a benchmark in between a CNN and SVR model, with all the benefits of a neural network in an architecture that was most like SVR. The network architecture consisted of one linear layer followed by a ReLU layer, mimicking the linear regression combined with a non-linear mapping function in SVR. In this model, all nodes in the first layer connected to all nodes in the second layer, resulting in a FNN.

The same pre-processing of the images was used as in the SVR method in order to adhere to the idea of mimicking SVR in a neural network: volume correction was performed first, followed by feature selection, and converting images into a one-dimensional array. Because of the nature of fully connected networks, the weights of the model reflect the contribution of specific voxels to the model’s prediction. Therefore, the model’s weights were used as attribution maps.

#### 2.3.4 Neural network implementation and training details

Models were implemented using PyTorch and experiments were run on one Nvidia Titan X Pascal GPU. An AdamW optimizer was used during training and a batch size of two was used.Training lasted for a total of 200 epochs. A learning rate of 5 × 10^−5^ was used in all simulation experiments, whereas a learning rate of 1 × 10^−4^ in combination with a reduce on plateau learning rate scheduler was used in the application on real cognitive data. The mean squared error (MSE) was used as a loss function in simulation experiments. This loss function was extended in the application on real cognitive data by including L1 for further regularization, resulting in loss=2∗MSE+L1.

### 2.4 Experiments

Three experiments were conducted using simulated cognitive datasets, a different dataset for each experiment. Simulated cognitive scores were computed based on the overlap of the WMH maps and predefined regions of interest (ROIs). The ROIs served as ground truth locations to be identified by the LSM methods, allowing evaluation of the accuracy of strategic lesion identification. As a final step in all variants of simulated cognitive datasets, min–max normalization was applied to normalize scores using values within the study population so that 1 corresponded to the highest score in each dataset.

#### 2.4.1 Experiment 1: Basic cognitive simulation

**Fig. 2:**
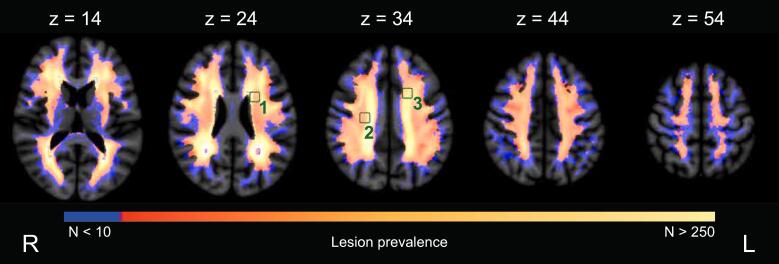
**WMH prevalence map (n = 821 patients) and the simulated ROI locations in green** Axial slices are indicated by z. (For interpretation of the references to colour in this figure legend, the reader is referred to the web version of this article.)

A WMH prevalence map was computed by summing all WMH maps of all participants in MNI-152 space. Three 10 × 10 × 10 mm^3^ ROIs were defined within the WMH prevalence map, similar to Zhang et al. (2014), see Fig. 2. ROI locations were determined based on the following two constraints: every voxel in the ROI should have WMH in ten or more patients and ROIs should not overlap. From all potential ROI locations, three were chosen randomly and were consistently used for all participants in the three simulation experiments. The centers of ROIs 1, 2, 3, were located in axial slice 24, 35, and 36 of MNI-152 space. Lesion load of an ROI for a patient was determined by the number of lesion voxels in the ROI normalized by the total number of voxels of that ROI. To obtain the final individual basic simulated cognitive scores, lesion load for the three ROIs was summed and, because no patient’s WMH overlapped with all ROI voxels, values were subsequently min–max normalized between 0 and 1 using the minimum and maximum values in the dataset. Hence, a higher simulated score was associated with a higher lesion load in the ROIs. Positive values in the attribution maps therefore indicated the positive contribution of lesion locations to the predicted scores. In section D of the Supplementary Materials, these experiments were repeated with six ROIs to study the effect of varying lesion prevalence.

#### 2.4.2 Experiment 2: Simulation with modified regional weights

A location in the brain that affects a cognitive domain more than other contributing locations should be identified by LSM methods. To investigate the methods’ capabilities to do so, a specific lesion location was weighted more heavily in its contribution to the simulated cognitive scores in the second experiment. The same strategy as described above was used to obtain the simulated cognitive data with modified regional weights. However, instead of equally summing the lesion loads of all three ROIs, they were multiplied by weights {w_1, w_2, w_3} corresponding to ROI 1, ROI 2, and ROI 3, respectively (see Eq. (2). In our experiments, the weights were set to {1,2,1} and {1,4,1}, weighing the contribution of the lesion load of ROI 2 more than those of the other ROIs.

The weighted score for patient *i*, with LL_ROI being the lesion load in a specific ROI, is defined as:

(2) $${weighted\_score}_{i}=\sum_{k=1}^{3}w_{k}{LL\_ROI}_{i,k}$$

#### 2.4.3 Experiment 3: Simulation with implemented noise

To compare the robustness of the three LSM methods to noise, basic simulation scores were perturbed by adding Gaussian noise. The Gaussian noise had a mean of 0 and standard deviation equal to the standard deviation of the original simulated score, referred to as *noise* in equation (3). Three different levels of noise were used, referred to as noise fraction *nf* in equation (3): 0.25, 0.50, and 0.75.

The perturbed score for patient *i* is defined as:

(3) $${noise\_score}_{i}=\left(1-{\mathrm{nf}}_{j}\right){basic\_score}_{i}+{\mathrm{nf}}_{j}∙{\mathrm{noise}}_{i}$$

#### 2.4.4 Experiment 4: Real attention and executive functioning scores

The relation between WMH and the real attention and executive functioning scores was analysed in a proof-of-principle experiment. Performance of the CNN with enhanced regularization was compared to the two benchmark models, SVR and the FNN. The same experiment was repeated for two neuropsychological tests that contribute to the attention and executive functioning, see section E of the Supplementary Materials.

### 2.5 Evaluation

#### 2.5.1 Predictive performance

All four experiments were performed in a 5-fold cross-validation. For every fold, a different 20 % of the data was used as test set, while the remaining 80 % was used to fit/train the models. For every patient in the test set, cognitive scores were predicted. This was repeated for the complete dataset over the course of 5 folds. Predictive performance was assessed for each fold by computing the R^2^ obtained through an ordinary least squares between the predicted and ground truth cognitive scores in the test set. In addition, the total WMH lesion volume was correlated with the cognitive scores to obtain a frame of reference. This process was the same for simulated and real cognitive data.

#### 2.5.2 Quantitative assessment attribution maps

In the basic simulation experiment and the experiment with added noise, group attribution maps were quantitatively evaluated using the precision-recall area under the curve (AUC). The three ROIs defined to generate the simulated cognitive data were used as ground truth locations. Descending threshold values between the maximum and minimum attribution values in the attribution maps were applied to create a binary image for which true and false positives as well as true and false negatives were determined. Precision and recall curves were not determined in the experiment using simulations with modified regional weights, because the ROIs did not contribute equally to simulated cognitive scores. Attributions in ROIs with less contribution are theoretically also lower in attribution value, skewing the precision-recall curves.

In addition to the precision-recall curves, attribution values within the ROIs were compared to attribution values outside the ROIs. This attribution ratio indicates whether the highest attribution values are concentrated in the ROIs. The higher the ratio, the more concentrated high attributions are located within the ROIs. The same analyses were repeated for a dilated version of the ROIs, for which an additional area of 5 mm surrounding the ROIs was considered ‘inside’ the ROIs. In case false positive attributions with a high value were located within 5 mm of the ROIs, the ratios would substantially increase. Attributions above 10 % of the maximum attribution value were taken into account for these analyses. Attribution ratios were determined for all simulation experiments.

Dice similarity coefficient and false negative ROIs were also determined. Accuracy of the attribution maps acquired using real cognitive data was not evaluated because there are no ground truth strategic lesion locations for real data.

#### 2.5.3 Qualitative assessment attribution maps

Attribution maps obtained in the application on real attention and executive functioning cognitive scores were assessed visually.

## 3 Results

### 3.1 Experiment 1: Model performance with basic cognitive simulations

Total WMH lesion volume had an R^2^ of 0.725 ± 0.065 with the simulated cognitive scores. This high value is because of the fact that the simulated cognitive scores were directly derived from the WMH volume in the three ROIs. True association between total WMH volume and real cognitive scores is usually low (see also Experiment 4 in section 3.4.); but for this technical validation study using simulated data this is not of concern.

SVR, the FNN, and the CNN were used to predict the basic simulated cognitive scores. Predictive performance of all three methods is provided in Table 1. The average predictive performance of the CNN across folds was 0.964 ± 0.008. For SVR and the FNN, predictive performances were 0.875 ± 0.019 and 0.863 ± 0.027, respectively.

**Table 1:** **Basic cognitive simulations: predictive performance and attribution map accuracy** For each method, predictions (N = 821 patients) were obtained from 5-fold cross-validation and the R^2^ averaged across folds. Precision-recall areas under the curve (AUC) were computed on the 5-fold mean (positive) group-level attribution map (see Fig. 3). Predictive performance was highest for the CNN, while the precision-recall AUC for the CNN was lowest. SVR and the FNN obtained similar predictive performance and precision-recall AUCs to one another. The ratio between attribution values inside and outside the ROIs was highest for the CNN. SVR and the FNN achieved a much lower ratio, but were comparable to one another. The same can be observed when ROIs were dilated with 5 mm. SD = standard deviation. ROI = region of interest. SVR = support vector regression. FNN = fully connected network. CNN = convolutional neural network.

	Predictive performance in R^2^ *Mean (SD)*	Precision-recall AUC	Ratio attributions inside vs. outside ROIs (factor increase with 5 mm ROI dilation)
SVR	0.875 (0.019)	0.693	0.13 (3.0x)
FNN	0.863 (0.027)	0.718	0.09 (2.3x)
CNN	0.964 (0.008)	0.625	1.02 (17.1x)

**Fig. 3:**
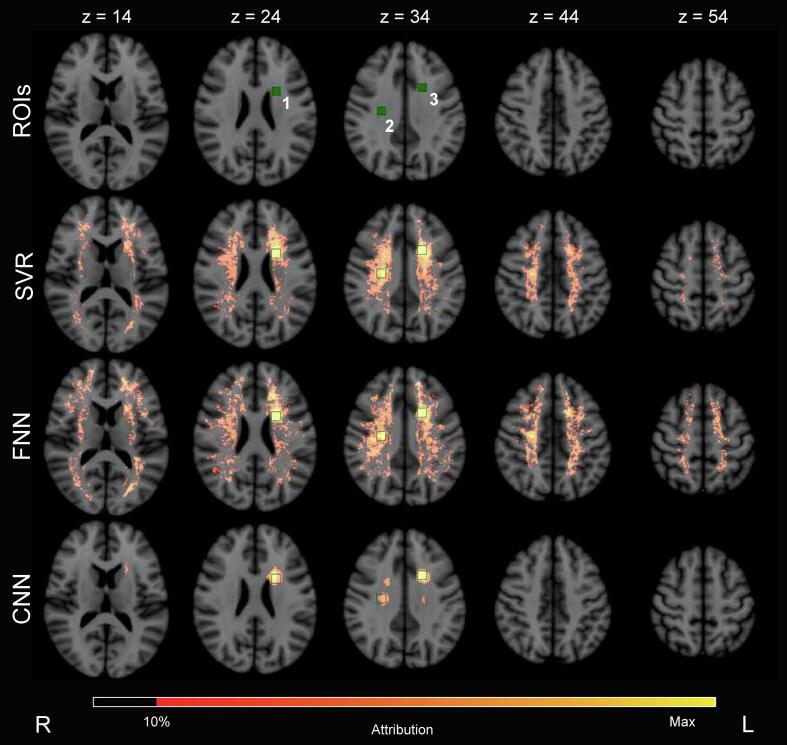
**Basic cognitive simulations: group-level lesion-symptom mapping results** ROIs were the ground truth locations for the cognitive simulations. Equal weight was assigned to the three ROIs. Positive group-level attribution values are visualized on five axial slices of MNI-152 space, indicated by z. It can be seen that all three methods assigned the highest attribution values inside of the three ROIs. However, the CNN with XAI was able to much better localize the three ROIs and had less false positive attributions more distant from the ROIs. SVR and the FNN had more false positive attributions further away from the three ROIs. Nevertheless, the precision-recall AUC (Table 1) was slightly better for SVR and the FNN, since the false attributions were of relatively low value; whereas the CNN with XAI had stronger attributions just outside the ROIs that results in a somewhat lower precision-recall AUC. SVR = support vector regression. FNN = fully connected network. CNN = convolutional neural network.

Precision-recall AUC of the 3D lesion attribution maps was highest for the FNN, followed by SVR, and the CNN; as can be seen in Table 1 and Figure S-1 in the Supplementary Materials. The group-level attribution maps are provided in Fig. 3. A standard deviation map for the CNN is provided in Figure S-2 of the Supplementary Materials. For all models the false positive voxels with the highest values in the attribution maps were predominantly directly adjacent to the ROIs. Compared to the other methods, the CNN had fewer false positive voxels more distant from the ROIs. Hence, the relatively low AUC for the CNN was primarily because of voxels with high attribution values directly outside the ROIs. Although in the basic simulation all ROIs had equal weight, attribution values varied between ROIs, with ROI 3 having the highest values. This partly reflects differences in the relative burden of WMH in the ROIs (mean percentage of ROI affected by WMH: 1: 8.60 %; 2: 6.23 %; 3: 8.23 %).

The CNN had the highest ratio between attribution values in- and outside the ROIs, see Table 1. Increase in the ratio when ROIs were dilated with 5 mm was also highest for the CNN. Figure S-3 in the Supplementary Materials provides an overview of the attribution locations that were taken into account for these analyses (threshold of 10 % of the maximum attribution value for each method).

Dice similarity coefficient and false negatives are reported in the Supplementary Material. The CNN achieved the highest Dice similarity coefficient results, consistent with the attribution ratio. No false negative ROIs were detected in group-level attribution maps.

**Fig. 4:**
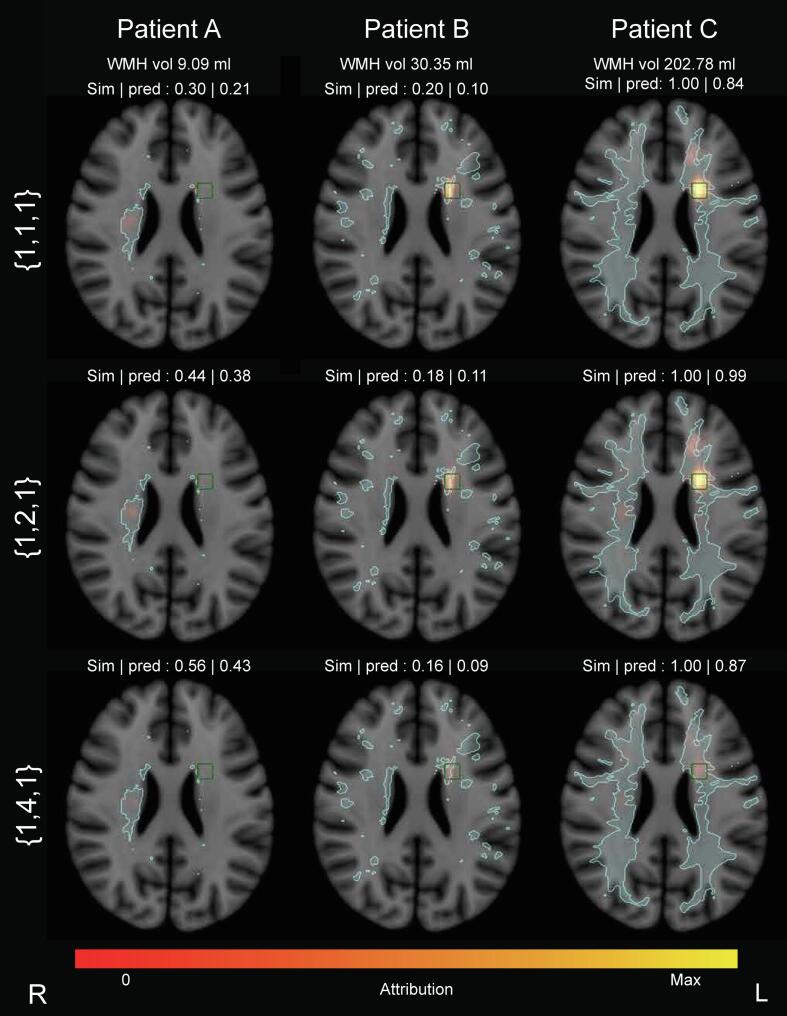
**Basic cognitive simulations and simulation with modified regional weights: individualized lesion-symptom mapping results obtained by the CNN with XAI** Examples of individual attribution maps for three simulated cognitive scores projected on axial slice 34 (containing ROI 2 right, ROI 3 left) of the MNI-152 template. WMH volume, simulated cognitive scores and predicted scores for each patient are denoted in the image. Simulated cognitive scores were based on WMH lesion load within the three ROIs. All scores were valued between 0 and 1, where 1 related to a higher lesion load in the ROIs. WMH lesion contours are displayed in light blue, the predefined ROI contours in green. TOP ROW: For the basic cognitive simulations, lesion load in all three ROIs was weighted equally: {1,1,1}. It can be seen that the CNN with XAI correctly highlighted the ROIs that contained lesions and no attributions were present in locations without lesions. In patient C, who had lesions in ROI 2 and 3, the attributed weight of ROI 3 was high, similar to the group results (Fig. 3); in patient A who only had lesions in ROI 2 this ROI had a higher attribution. MIDDLE AND BOTTOM ROWS: Here, lesion load in ROI 2 was weighted more heavily in the simulations than the other ROIs, depicted in row 2 {1,2,1} and 3 {1,4,1}. This yielded different simulated cognitive scores. Patient A had a white matter lesion that overlaps clearly with ROI 2, but not with the other ROIs. Different weightings of this ROI in the simulations therefore had little effect in this patient. Patient B had very little WMH in either of the ROIs presented in the figure and no prominent attribution can be seen in the lesions in locations outside the ROIs. Figure S-4 in the supplementary materials shows that most of the attribution could be found in ROI 1 on axial slice 24 of MNI-152 space. With increasing ROI 2 wt, the small WMH in ROI 2 received higher attribution values. Patient C had the highest WMH volume in this example, the lesion covered a substantial portion of the brain and overlapped with ROIs almost completely, resulting in a score of 1.00 regardless of ROI weights. With equal weighting of the ROIs, the most prominent attribution were found in ROI 3. With increased weighting of ROI 2 in the simulations, the attribution values in ROI 2 increased with higher weight relative to ROI 3. WMH vol = white matter hyperintensity volume. Sim = simulated cognitive score. Pred = predicted cognitive score. (For interpretation of the references to colour in this figure legend, the reader is referred to the web version of this article.)

Fig. 4 presents examples of individual attribution maps for the CNN with XAI, for three patients with varying WMH volumes and simulated cognitive scores. It can be seen that the CNN with XAI method correctly highlighted the WMH in the ROIs, and did not assign considerable contribution to the other WMHs present in these participants. When the relative weight of ROI 2 was increased, this was directly reflected in increased attribution values measured at this ROI; suggesting that the CNN with XAI was able to capture these differences between ROIs. Some attribution values fell outside the ROIs, but were in close proximity or of substantially lower value. In case no WMH was present within an ROI, that location was not highlighted.

### 3.2 Experiment 2: Model performance with modified regional weights

The basic simulation of cognitive data were altered by increasing the contribution of ROI 2 twice, while retaining the same contribution weight for ROIs 1 and 3, i.e.: {1,2,1} and {1,4,1}.

Just as in the analysis using basic cognitive simulations, predictive performance for all three models was high, see Table S-3 in the Supplementary Materials, with the highest R^2^ obtained by the CNN for both datasets with modified regional weights.

**Fig. 5:**
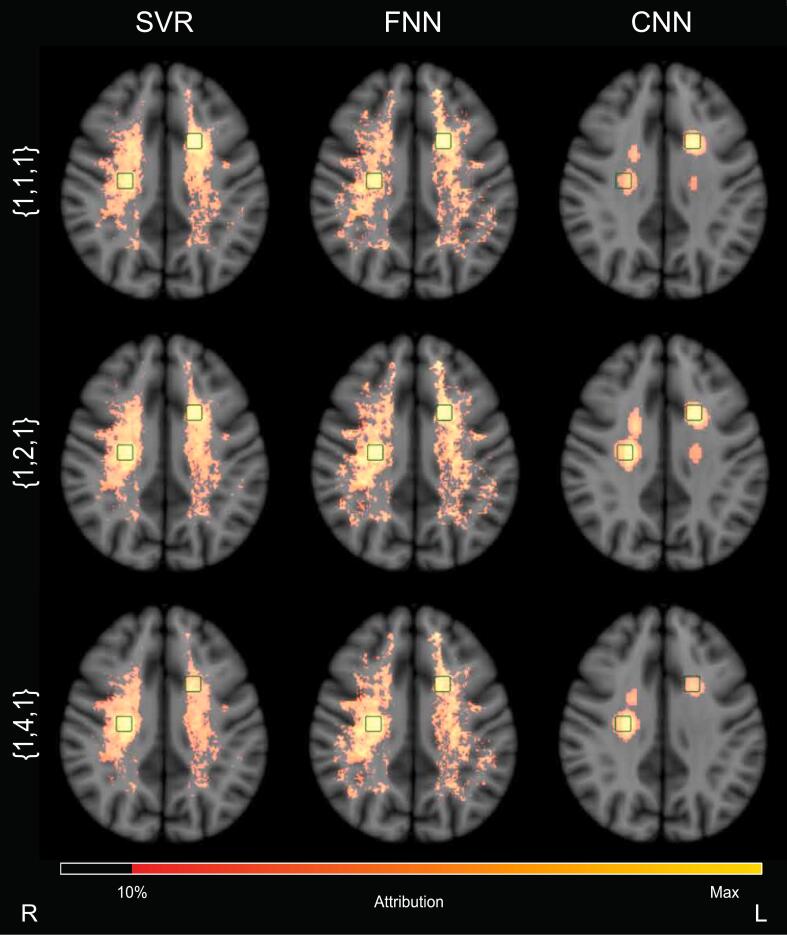
**Simulation with modified regional weights: group-level lesion-symptom mapping results** Positive group-level attribution values for each method are visualized on axial slice 34 of MNI-152 space. Contours of ROI 2 (right side of the brain) and 3 (left side of the brain) are displayed in green. Each row corresponds to a different set of ROI weights. TOP ROW: All ROI contributions to the simulated cognitive score were weighted equally, as shown previously in Fig. 3. MIDDLE AND BOTTOM ROWS: The contribution of ROI 2 to the simulated cognitive score was weighted stronger than the other ROIs. The columns correspond to the three different methods. It can be seen that for all methods, attribution values in ROI 2 increased with higher contribution. This is most prominent for the CNN with XAI. The number of false positive attributions appeared consistently lowest for the CNN with XAI. SVR = support vector regression. FNN = fully connected network. CNN = convolutional neural network. XAI = explainable artificial intelligence. (For interpretation of the references to colour in this figure legend, the reader is referred to the web version of this article.)

Group level attribution maps for each method are displayed in Fig. 5. With increasing weighing of ROI 2′s contribution, its attribution visibly increased for all methods. The same outcomes were achieved in the individual attribution maps presented in Fig. 4. With the modified weights, the amount of false positive voxels distant from the ROIs in SVR and the FNN remained essentially the same.

Table 2 shows the ratios between attribution values in- and outside the ROIs. Similar to the experiment with basic cognitive simulations, the CNN achieved the highest ratio and highest increase with dilated ROIs.

**Table 2:** **Simulation with modified regional weights: ratio between attribution values inside and outside ROIs** Factor of increase with ROIs dilated with 5 mm provided in parenthesis. Ratio of attribution values inside and outside the ROIs was highest for the CNN. Lower, comparable ratios were found for SVR and the FNN. A similar pattern can be observed in increase factors for dilated ROIs. SVR = support vector regression. FNN = fully connected network. CNN = convolutional neural network.

	Modified regional weights configuration	
	{1,2,1}	{1,4,1}
SVR	0.12 (3.1x)	0.13 (3.3x)
FNN	0.08 (2.5x)	0.07 (2.7x)
CNN	0.69 (8.4x)	0.93 (17.2x)

### 3.3 Experiment 3: Model sensitivity to noise

Three levels of noise were added to the simulated cognitive data to test the robustness of the three models. With a noise level of 0.25, 0.50, and 0.75, the R^2^ of total WMH lesion volume alone as regressor for the simulated cognitive scores was 0.651, 0.395, and 0.062, respectively. Of note, the explained variance of total WMH lesion volume (see Experiment 4) for the real attention and executive functioning cognitive scores was 0.013.

**Fig. 6:**
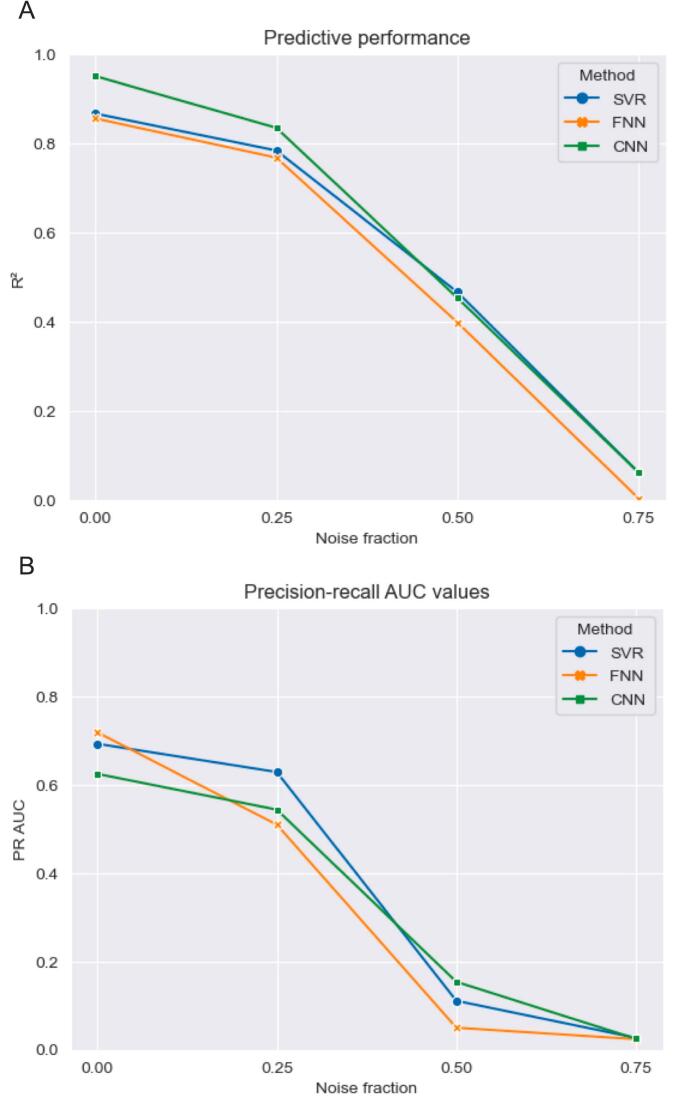
**Effect of noise on model performance** Predictive performance in R^2^ (A) and precision-recall AUC (B) for each LSM method for different noise fractions (0 – 0.75). R^2^ was determined by concatenating the predictions made in the cross-validation and evaluating them against the artificial cognitive scores using an ordinary least squares. Precision-recall AUC were computed on the (positive) group-level attribution map for each method. Methods showed a similar decline in predictive performance and precision-recall AUCs with increasing noise fractions. PR AUC=Precision-recall area under the curve. SVR = support vector regression. FNN = fully connected network. CNN = convolutional neural network.

Changes in predictive performance and precision-recall AUC as a result of noise are visualized in Fig. 6. Values can also be found in Table S-4 and S-5 of the Supplementary Materials. Overall, performance of all methods for both metrics dropped because of increasing noise. The three models display a similar decline pattern. Attribution values inside the ROIs compared to outside the ROIs, see Table 3, decreased with noise. This decrease was most prominent for the CNN. The factors of ratio increase with dilated ROIs was relatively consistent with varying noise factors for SVR and the FNN. At a noise fraction of 0.75, the factor for the CNN increased substantially, coinciding with low attribution ratios for the original ROIs.

**Table 3:** **Model sensitivity to noise: ratio between attribution values inside and outside ROIs** Factor of increase with ROIs dilated with 5 mm provided in parenthesis. Ratios between attribution values inside and outside the ROIs decrease with noise. For noise fractions 0.25 and 0.50, the CNN obtained the highest ratios and factor increase with ROIs that were dilated with 5 mm. SVR achieved the second highest ratios, closely followed by the FNN. SVR = support vector regression. FNN = fully connected network. CNN = convolutional neural network.

	Noise fraction		
	0.25	0.50	0.75
SVR	0.12 (2.9x)	0.07 (3.2x)	0.03 (3.7x)
FNN	0.07 (2.3x)	0.04 (3.1x)	0.03 (3.4x)
CNN	0.60 (5.4x)	0.17 (3.0x)	<0.01 (323.4x)

### 3.4 Experiment 4: Proof of principle on real world cognitive data

As a proof-of-principle demonstration, the methods were applied to predict actual norm adjusted attention and executive functioning scores based on WMH maps (Table 4). SVR achieved the highest predictive performance with a mean R^2^ of 0.291 across folds, followed by the CNN with a mean R^2^ of 0.216 and the FNN with a mean R^2^ of 0.020. Variance between cross-validation folds was highest for the CNN and smallest for the FNN. The R^2^ of total WMH lesion volume alone was 0.013, meaning both the SVR and CNN achieved a much better predictive performance.

**Table 4:** **Proof of principle on real attention and executive functioning scores: predictive performance** Predictions (N = 813) made in the cross-validation were combined and evaluated against the real attention and executive functioning cognitive scores using the R^2^ obtained through an ordinary least squares. SVR = support vector regression. FNN = fully connected network. CNN = convolutional neural network.

	Predictive performance in R^2^
SVR	0.291 (0.038)
FNN	0.020 (0.012)
CNN	0.216 (0.063)

**Fig. 7:**
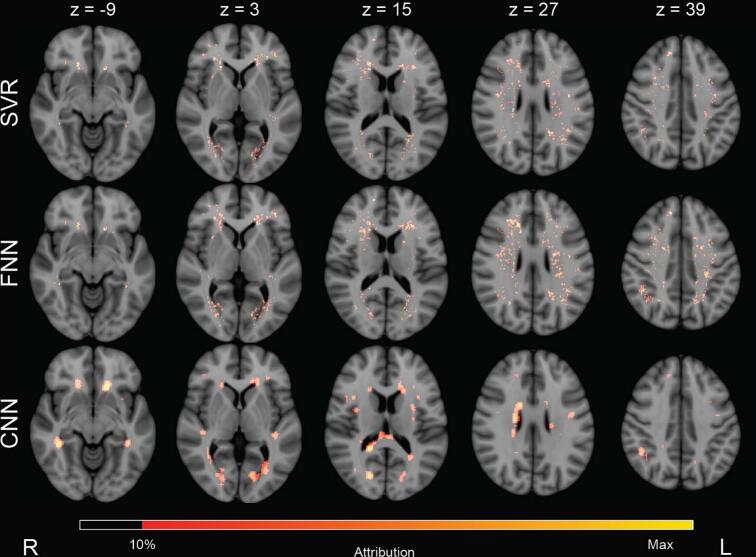
**Proof of principle on real attention and executive functioning scores: group-level lesion-symptom mapping results** Positive group-level attribution maps visualized on five axial slices of MNI-152 space for all three models. Axial slices are indicated by z. SVR and the FNN highlighted more dispersed locations and the attributions were localized. The CNN with XAI highlighted larger locations, which may be explained by the sliding window principle on which the XAI method (occlusion) is based. When a location of high importance is determined, the whole location of the cubic window will receive attribution values. This also explains attribution values in non-white matter areas. Overall, all three methods appeared to have their attributions located in roughly the same parts of the brain and seem to be in agreement. Since there is no ground truth on the actual associations with the real cognitive scores, this is a qualitative assessment. SVR = support vector regression. FNN = fully connected network. CNN = convolutional neural network.

The group-level attribution maps of all methods are shown in Fig. 7. Since no ground truth of actual strategic lesion locations can be established for real cognitive data, maps were compared visually. The group-level attribution maps obtained by SVR and the FNN show similar patterns. The group-level attribution map of the CNN with XAI differs more, but it does show overlapping locations with SVR and the FNN. White matter tracts with the highest attribution values can be found in Table S-9 of the Supplementary Materials.

**Fig. 8:**
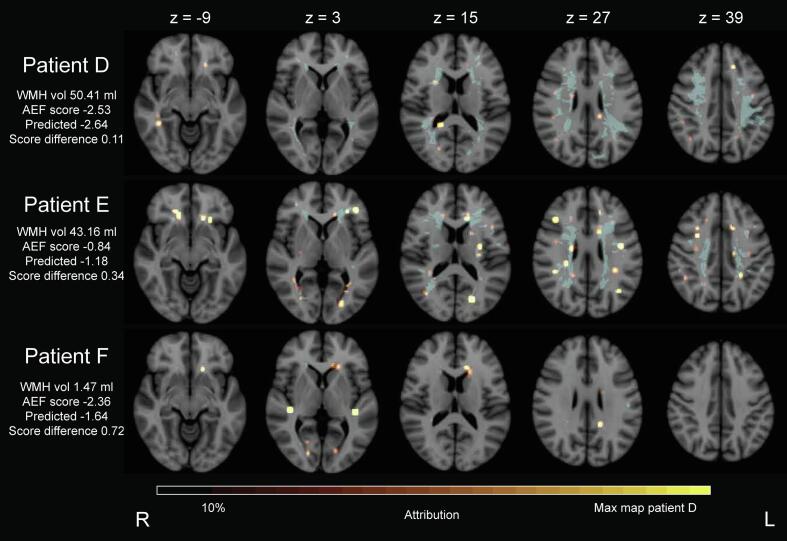
**Proof of principle on real attention and executive functioning scores: individualized lesion-symptom mapping results obtained by the CNN with XAI** Three patient examples of individual attribution maps. Patients were selected based on their WMH volume and attention and executive functioning score. Patient D has a high WMH volume and a large negative Z-score, while patient E has a small negative Z-score and a high WMH volume. Patient F has a low WMH volume with a large negative Z-score. WMH volume for each patient is denoted in the image, as well as their norm corrected attention and executive functioning Z-score, the predicted score, and the difference between measured and predicted score. WMH lesions are depicted in blue. Colour bars were based on the attribution map of patient D, which contained the highest attribution value among the selected patients. Because this illustrative colour bar was set based on the attribution map with highest values, attribution maps can be compared between patients. For all patients, specific locations within the WMH map were highlighted. The highlighted locations mostly differ among these patient examples. The model’s score prediction was best for patient D, with the smallest difference between true attention and executive functioning score and predicted score, whereas patient F had the worst prediction. Patient F had a low WMH volume, combined with a substantially impaired attention and executive functioning score. The chance that the WMH map would explain the score was therefore low. This combination of low WMH and bad score is an indication that the cognitive performance of this patient is at least partly explained by information other than the WMH map. WMH vol = white matter hyperintensity volume. AEF = attention and executive functioning. (For interpretation of the references to colour in this figure legend, the reader is referred to the web version of this article.)

Individual attribution maps for three patients with varying combinations of WMH volume and attention and executive functioning scores can be found in Fig. 8. Again, these analyses of the attribution maps are qualitative, because no ground truth data exists. Since the predictive performance of the methods is low, caution should be paid when inspecting these attribution maps.

## 4 Discussion

In this study, we introduced a CNN combined with XAI to perform lesion-symptom mapping for white matter hyperintensities. The CNN showed comparable results to SVR and the FNN benchmarks in three simulation experiments. The added benefit is that it was able to generate patient-specific lesion attribution patterns. When applied to real data, the predictive performance of the CNN was considerably higher than for WMH total volume alone, but not superior to SVR. Overall, these results support the potential application of neural network technology with XAI for lesion-symptom mapping.

When comparing results obtained by SVR and the CNN more specifically, predictive performance of the CNN consistently surpassed that of SVR in experiments using simulated cognitive data. On real attention and executive functioning scores, SVR obtained somewhat better predictive performance than the CNN; but it should be noted that overall R^2^ was low (<0.3). In the simulation experiments, both methods showed high attribution values in the predefined ROIs, while more variation was observed between attribution maps obtained with real cognitive data.

Our proposed method creates patient-specific attribution maps, whereas conventional LSM methods only establish the relationship between brain lesion and cognitive score on a group level (Seghier and Price, 2023, Moore et al., 2024). It should be noted that attribution maps obtained for an individual patient, such as in Fig. 8, should always be regarded within the context of that specific patient. Lesion locations that contribute to a higher predicted score (i.e. worse cognitive performance) receive higher attribution values, regardless of the predicted cognitive score. This means that individual attribution maps highlight locations that explain why an impaired cognitive performance was predicted, not why a cognitive score was predicted in general. Therefore, these individual attribution maps are of value when the predicted score indicates similar or worse cognitive ability than the measured cognitive ability. When a predicted cognitive score indicates a better cognitive performance than what was measured, the analysed lesion (in this case, WMH) likely does not explain the cognitive decline and the attribution maps are less likely to contain relevant information. In those cases, other pathology that was not included in the analysis, such as infarcts and amyloid β deposition, may play a role in the impaired cognitive ability. In patients for whom a worse cognitive performance was predicted than what was measured, the patient likely has a relatively high cognitive reserve compared to other patients in the dataset. But before the individual attribution maps can be used in clinical research or routine, several aspects need to be addressed. For instance, validation of the method on different data is necessary. Moreover, a model’s certainty score will be required for each patient to provide clinicians with an indication of the potential value of attribution maps. The proposed method of a CNN with XAI could be potentially extended in the future to include multiple types of pathology and an uncertainty estimate of the model’s prediction, further supporting the interpretation of individual results.

In general, validation of attribution maps obtained with XAI is complex (Nauta et al., 2023). In this study, we used simulated cognitive data that enabled the evaluation of attribution maps qualitatively and quantitatively using the precision-recall AUC based on the overlap with the predefined ROIs. Nevertheless, the point at which attribution maps can be considered good enough remains ambiguous. In the precision-recall AUC, methods are penalized when attribution values are outside of the ROIs, regardless of distance to the ROI. It could be questioned whether a difference in a few voxels is clinically relevant and alternative evaluation metrics are part of future research in the XAI community. Ratios between attribution values in- and outside the ROIs provided insight into how concentrated attribution values were located in the ground truth locations. By also taking into account the factor increase of this ratio when ROIs were dilated with 5 mm, information about attribution values extending just beyond the ROI boundaries was considered. This shows that the CNN has false positive attributions just outside the ROIs, whereas the SVR and FNN approach have much more widespread false positives throughout the brain. False negative ROIs do not appear to be an issue with all approaches.

Evaluating LSM methods without ground truth strategic lesion locations is challenging. Methods can be compared to each other, but a higher explained variance might not always indicate a better strategic lesion identification. Therefore, simulated cognitive data were used to provide ground truth locations for extensive evaluation and controlled experimentation. However, by design, the simulated scores were highly correlated with total WMH volume, which does not reflect the real relation between WMH and cognitive scores. Cognitive performance in patients is also influenced by other vascular lesion types and factors such as amyloid β deposition in the brain and educational background. The simulations were kept simple to demonstrate basic functioning of this novel XAI-based method for LSM. In the simulation experiment with noise, more realistic correlations between WMH volume and scores were achieved. It should be noted that the noise in this experiment was random, which is not the case in real patients. To overcome the limitations of the simulated data, the proposed method was also tested on real attention and executive functioning scores.

The simulation with modified regional weights, inspired by Zhang et al. (Zhang et al., 2014), was implemented with the idea that lesions in one location can have a bigger effect on a cognitive domain than other locations. For instance, an infarct located in the Broca area will have more effect on verbal memory than an infarct located in the motor cortex. However, it is unclear whether this holds true for WMH. The experiment nonetheless allowed for testing whether the various LSM approaches could extract the non-uniform contributions to the simulated scores. Visually, all methods assigned higher attribution values to the location with increased contribution to the simulated cognitive scores. The change in attribution maps as a result of modified regional weights was most prominent for the CNN with XAI.

A sufficient amount of data is required to apply LSM methods. Studies have been conducted to test the appropriate sample size for SVR, where datasets of 100–120 subjects seem sufficient and performance plateaus with larger datasets (Sperber et al., 2019). Neural networks are known to require substantially larger dataset sizes and show increasingly better performance with more data. Over time, more data, information, and brain coverage has been acquired for LSM studies by pooling data and creating large multi-center cohorts (Weaver et al., 2021, Coenen et al., 2023). These datasets can be used to further validate the use of neural networks for LSM with the potential prospect of achieving increasingly better results, potentially outperforming SVR. In this study, attribution values in ROI 2 were substantially lower without modified regional weights, likely because of reduced lesion prevalence, suggesting that more data might improve the model further. In the additional experiments in section D of the Supplementary Material, we observed that the attribution maps computed using the CNN with XAI were more sensitive to this effect than SVR and the FNN. However, predictive performance was more stable for the CNN when ROIs were in low prevalence locations. Effects of varying lesion prevalence will be included in future research.

A CNN with XAI might not always be the best LSM option. Once a neural network has been trained, it can be applied to smaller datasets as long as the training data has a similar distribution as the new data, e.g. lesion distribution and volume, patient age, sex, level of education, and diagnosis. But when a small dataset needs to be analysed and no trained model is available, or the training data characteristics do not match the test data, a neural network-based approach might not be ideal. Other methods such as mass-univariate, SVR, or sparse canonical correlation analysis for neuroimaging may be better suited (Bates et al., 2003, Rorden et al., 2007, Zhang et al., 2014, Pustina et al., 2018). In case computational power is limited, a neural network-based approach might be less convenient than alternatives. However, in case individualized information is beneficial, large datasets and computing power are available, a CNN with XAI might be a fitting LSM method.

This study has several limitations. First, various XAI methods are available that could be used in this study but only occlusion was used here (van der Velden et al., 2022). The occlusion method is stable and results in attribution maps with little noise, but it is very slow. Choosing a different method is a trade-off between speed and crisp, stable attribution maps. It should also be noted that a shift in high attribution values of a few voxels with respect to the ROI cubes could be observed in the attribution maps obtained with the XAI method. This might be because occlusion relies on a sliding window principle to compute the attribution. Convolution layers in the CNN itself might also contribute to the shift because they rely on a similar sliding window. Second, as previously mentioned, data is an important factor when using neural networks. For training and testing a neural network, 821 subjects is a small number. Future work could be conducted to apply the proposed method on other and larger datasets to further validate the presented LSM approach. In terms of the data itself, WMH were chosen as lesion type to develop the new LSM method. A downside of using WMH instead of infarcts is the fact that there is a weaker correlation between WMH lesions and cognitive outcomes. Applications on infarct data might be of value as well, since the association between infarct location and cognitive scores is stronger than for WMH and more LSM studies have been conducted on infarcts previously (de Haan and Karnath, 2018). It should also be noted that WMH have a high prevalence and can cover a relatively high brain volume (Duering et al., 2023). Vascular lesions such as cerebral microbleeds and perivascular spaces are much smaller, ≤10 mm and ≤2 mm respectively (Duering et al., 2023). LSM using those smaller lesions poses an additional need for large datasets to achieve substantial brain coverage, or a different approach such as a regional-based LSM technique. Third, in this study we only considered relatively simple CNN architectures. However, this study is intended as a proof of principle for the use of neural networks combined with XAI for LSM. The results warrant further study of different neural networks. Fourth, feature selection was used in the proof-of-principle experiment. This step was necessary to achieve a substantial model performance by reducing the input complexity using prior knowledge of a mass-univariate analysis. In the future, an end-to-end solution without feature selection would be preferred to prevent potential bias of the mass-univariate analysis. Finally, patients with cognitive decline often exhibit multiple vascular lesions in the brain simultaneously. These lesions can have a cumulative impact and should therefore be accounted for (Heinen et al., 2018). Additionally, cognition may be impaired in multiple domains, which can be correlated and have both distinctive and shared strategic lesion locations (Sperber et al., 2020, Weaver et al., 2023). The current work was limited to a single input and a single output, only enabling the analysis of a single lesion type and single cognitive score at a time. Expanding the neural network-based LSM method to incorporate supplementary patient information, such as additional vascular brain lesions, disconnection information, non-lesion related brain changes, fluid biomarkers, and risk factors may be a valuable direction for future work.

## 5 Conclusion

The use of a neural network combined with eXplainable AI for lesion-symptom mapping was introduced and evaluated in this study. Results obtained using simulated and real cognitive data show the potential of this novel approach, with the added benefit of generating individualized attribution maps. Further validation and development of the method is necessary before it can be implemented in clinical research or routine. This technique could be expanded to account for multiple lesion types and multiple cognitive domains by altering the architecture of the CNN, pushing the boundaries of lesion-symptom mapping possibilities even further.

## 2.2 Data

### 2.2.1 Study population

Patients were included from the “Utrecht-Amsterdam clinical features and prognosis in vascular cognitive impairment (TRACE-VCI)” study population (Boomsma et al., 2017). In short, the aim of the TRACE-VCI study was to investigate the clinical features and prognosis of patients with possible VCI in a memory clinic setting. TRACE-VCI included 860 consecutive patients with evidence of vascular brain injury from Amsterdam University Medical Center (N = 664) and from two outpatient memory clinics of the University Medical Center Utrecht (N = 196). For the current study, only patients with available WMH segmentations and successful registrations in Montreal Neurological Institute (MNI) space (missing for N = 39) and cognitive test results for attention and executive functioning (missing for N = 8) were eligible, leaving a study population of 813 (Coenen et al., 2023). Median WMH volume was 8 ml and with an interquartile range of 3.2–20.9 ml. Mean age was 67.5 years (SD = 8.5 years) and 46.1 % were female. Further participant details in Supplementary Table S-1.

All patients presented with cognitive complaints at the clinics and were eligible for TRACE-VCI if their brain MRI showed evidence of vascular brain injury (i.e. Fazekas scale ≥ 2), ≥1 lacunar infarct(s), ≥1 non-lacunar (large vessel) infarct(s), ≥ 1 cerebral microbleed(s), ≥1 intracerebral hemorrhage(s) and/or mild WMH (Fazekas 1) with an increased vascular risk defined as the presence of ≥2 vascular risk factors (hypertension, hypercholesterolemia, diabetes mellitus, obesity, current smoking or a reported history of a vascular event other than stroke) (Boomsma et al., 2017). Patients were included regardless of objective cognitive severity, including patients with dementia (51 %), mild cognitive impairment (MCI) (25 %) and no objective cognitive impairment (24 %).

Each patient underwent a standardized extensive one-day memory clinic evaluation including an interview, physical and (cognitive) neurological examination, laboratory testing, standardized neuropsychological testing and an MRI-scan of the brain. The studies were approved by the medical ethics committee of Amsterdam University Medical Center and University Medical Center Utrecht. We obtained written informed consent from participants before conducting research-related procedures.

### 2.2.2 MRI data and processing

Images were acquired using 3D fluid-attenuated inversion recovery (FLAIR) sequences on a variety of scanners and using various parameters, as described previously by Boomsma et al. (2017) (Boomsma et al., 2017). The following scanners were employed: 1.5 T and 3.0 T GE Signa HDxt, 3.0 T GE Discovery MR750, as well as 3.0 T Philips Ingenuity. The number of acquired slices ranged from 128 to 321, voxel sizes were between 0.98–1.21 × 0.98–1.21 × 0.56–1.30 mm^3^. Repetition times, echo times, and inversion times differed between sequences. WMH were semi-automatically delineated on the MRI using the k-nearest neighbour classification with tissue type priors (kNN-TTP) method, with rigorous quality control and visual checks of all segmentations, as described by Groeneveld et al. (2019) (Groeneveld, 2019, Steenwijk, 2013). Segmentation masks were subsequently registered to the 1 × 1 × 1 mm^3^ MNI-152 brain template using Elastix for spatial normalization (Fonov et al., Jan. 2011, Klein et al., 2010).

### 2.2.3 Cognition data

Real attention and executive functioning scores were obtained by averaging norm-referenced Z-scores of the Trail Making Test part B, Digit Span Forward and Backward, semantic fluency (animal naming, 1 min), and phonemic fluency tasks (letters, 1 min) as described by Coenen et al. (2023) (Coenen et al., 2023). Scores were corrected for education, age, and sex using Dutch population-based normative data published on the website of The Dutch Association of Psychologists in 2012. Dutch normative data from the Wechsler Adult Intelligence Scale IV adjusted for age was used for the Digit Span tests. Descriptive statistics summarizing the individual neuropsychological test results are provided in Table S-4 of the Supplementary Materials by Coenen et al. (2023) (Coenen et al., 2023). The percentage abnormal neuropsychological test results can be found in Table S-2 of the Supplementary Materials. Finally, scores were normalized using min–max feature scaling using values of the study population, where a higher score is related to a worse cognitive performance.

Simulated cognitive scores were used in the first three experiments and were computed based on patients’ WMH volume in selected regions-of-interest, as described in more detail in Section 2.4.

## CRediT authorship contribution statement

**Ryanne Offenberg:** Writing – original draft, Methodology, Formal analysis, Conceptualization. **Alberto de Luca:** Writing – review & editing, Supervision, Conceptualization. **Geert Jan Biessels:** Writing – review & editing, Supervision, Funding acquisition, Data curation, Conceptualization. **Frederik Barkhof:** Writing – review & editing, Data curation. **Wiesje M. van der Flier:** Funding acquisition, Data curation, Writing – review & editing. **Argonde C. van Harten:** Writing – review & editing, Data curation. **Ewoud van der Lelij:** Writing – review & editing. **Josien Pluim:** Writing – review & editing, Supervision, Conceptualization. **Hugo Kuijf:** Writing – review & editing, Supervision, Project administration, Methodology, Funding acquisition, Conceptualization.

## Funding

This study received funding from the Dutch Heart Foundation (03-004-2021-T043). This work was also supported by TAP-Dementia, as funded project by ZonMw (#10510032120003) part of the Dutch National Dementia Strategy, for a Timely Accurate and Personalized diagnosis of dementia. TAP-dementia receives co-financing from Avid Radiopharmaceuticals, Roche diagnostics, and Amprion. The research of Alberto De Luca is also supported by funding from Alzheimer Nederland (WE-03-2022-11). Research of Alzheimer center Amsterdam is part of the neurodegeneration research program of Amsterdam Neuroscience. Alzheimer Center Amsterdam is supported by Stichting Alzheimer Nederland and Stichting Steun Alzheimercentrum Amsterdam. The chair of Wiesje van der Flier is supported by the Pasman stichting.

## Declaration of Competing Interest

The authors declare the following financial interests/personal relationships which may be considered as potential competing interests: Hugo Kuijf reports financial support was provided by Netherlands Heart Foundation. Geert Jan Biessels, Wiesje M. van der Flier reports financial support was provided by Netherlands Organisation for Health Research and Development. Frederik Barkhof reports a relationship with NIHR University College London Hospitals Biomedical Research Centre that includes: funding grants. Frederik Barkhof reports a relationship with Biogen that includes: board membership. Frederik Barkhof reports a relationship with Merck that includes: board membership and consulting or advisory. Frederik Barkhof reports a relationship with EISAI that includes: board membership. Frederik Barkhof reports a relationship with Prothena that includes: board membership. Frederik Barkhof reports a relationship with Combinostics that includes: board membership. Frederik Barkhof reports a relationship with Scottish Brain Sciences that includes: board membership. Frederik Barkhof reports a relationship with Alzheimer Europe that includes: board membership. Frederik Barkhof reports a relationship with Roche that includes: consulting or advisory. Frederik Barkhof reports a relationship with Celltrion that includes: consulting or advisory. Frederik Barkhof reports a relationship with Rewind Therapeutics that includes: consulting or advisory. Frederik Barkhof reports a relationship with Bracco that includes: consulting or advisory. Wiesje M. van der Flier reports a relationship with Netherlands Organisation for Health Research and Development that includes: funding grants. Wiesje M. van der Flier reports a relationship with Dutch Research Council that includes: funding grants. Wiesje M. van der Flier reports a relationship with EU-JPND that includes: funding grants. Wiesje M. van der Flier reports a relationship with Alzheimer Netherland that includes: funding grants. Wiesje M. van der Flier reports a relationship with Hersenstichting CardioVascular Onderzoek Nederland that includes: funding grants. Wiesje M. van der Flier reports a relationship with Health Holland that includes: funding grants. Wiesje M. van der Flier reports a relationship with Topsector Life Sciences & Health that includes: funding grants. Wiesje M. van der Flier reports a relationship with Dioraphte Foundation that includes: funding grants. Wiesje M. van der Flier reports a relationship with Gieskes-Strijbis Fund Foundation that includes: funding grants. Wiesje M. van der Flier reports a relationship with Stichting Equilibrio that includes: funding grants. Wiesje M. van der Flier reports a relationship with Edwin Bouw fonds that includes: funding grants. Wiesje M. van der Flier reports a relationship with Pasman stichting that includes: funding grants. Wiesje M. van der Flier reports a relationship with Stichting Alzheimer & Neuropsychiatrie Foundation that includes: funding grants. Wiesje M. van der Flier reports a relationship with Philips that includes: funding grants. Wiesje M. van der Flier reports a relationship with Biogen MA Inc that includes: board membership, consulting or advisory, and funding grants. Wiesje M. van der Flier reports a relationship with Novartis-NL that includes: funding grants. Wiesje M. van der Flier reports a relationship with Life-MI that includes: funding grants. Wiesje M. van der Flier reports a relationship with AVID that includes: funding grants. Wiesje M. van der Flier reports a relationship with Roche that includes: board membership, consulting or advisory, and funding grants. Wiesje M. van der Flier reports a relationship with Eli-Lilly-NL that includes: board membership and funding grants. Wiesje M. van der Flier reports a relationship with Fujifilm that includes: funding grants. Wiesje M. van der Flier reports a relationship with Eisai that includes: consulting or advisory and funding grants. Wiesje M. van der Flier reports a relationship with Combinostics that includes: funding grants. Wiesje M. van der Flier reports a relationship with Oxford Health Policy Forum CIC that includes: consulting or advisory. Co-author is member of the steering committee or data safety monitoring board member for Biogen, Merck, Eisai and Prothena. - FB Co-author has research agreements with ADDI, Merck, Biogen, GE Healthcare, Roche. - FB Co-author is co-founder and shareholder of Queen Square Analytics LTD. - FB Co-author holds the Pasman chair. - WF Co-author is recipient of ABOARD, which is a public-private partnership receiving funding from ZonMW (#73305095007) and Health Holland, Topsector Life Sciences & Health (PPP-allowance; #LSHM20106). - WF Co-author has been an invited speaker at Biogen MA Inc, Danone, Eisai, WebMD Neurology (Medscape), NovoNordisk, Springer Healthcare, European Brain Council. - WF Co-author was associate editor of Alzheimer, Research & Therapy in 2020/2021. - WF Co-author is associate editor at Brain. - WF Co-author is member of the steering committee of EVOKE/EVOKE+ (NovoNordisk). - WF Co-author is member of the steering committee of PAVE, and Think Brain Health. – WF. If there are other authors, they declare that they have no known competing financial interests or personal relationships that could have appeared to influence the work reported in this paper.

## Data Availability

TRACE VCI data can be made available upon request, within the boundaries of privacy and legal regulations.
