# Supplementary material for: Individualized lesion-symptom mapping using explainable artificial intelligence for the cognitive impact of white matter hyperintensities
Source: Neuroimage Clin. 2025 Apr 18;46:103790. doi: 10.1016/j.nicl.2025.103790 (PMC12047604; doi:10.1016/j.nicl.2025.103790)
Supplement: Supplementary Data 1 [file mmc1.docx]

Supplementary material

[Section A. Study population characteristics 1](#_Toc194066023)

[Section B. Supplementary Tables and Figures 2](#_Toc194066024)

[Section C. Additional analyses 7](#_Toc194066025)

[Dice similarity coefficient 7](#_Toc194066026)

[False negatives 7](#_Toc194066027)

[White matter tracts with the highest attribution values 9](#_Toc194066028)

[Section D. Additional experiments with simulated cognitive data 10](#_Toc194066029)

[Section E. Additional analysis on neuropsychological tests 14](#_Toc194066030)

# Section A. Study population characteristics

Table S-1. Study population characteristics
Data is missing for some patients, in that case the total N of available information is noted in the left column. Level of education is given according to the Verhage scale: (1) <6 years of primary education, (2) finished 6 years of primary education, (3) 6 years primary education and <2 years of low level secondary education, (4) 4 years of low level secondary education, (5) 4 years of average level secondary education, (6) 5 years of high level secondary education, (7) university degree [1]. SCI=Subjective Cognitive impairment. MCI=mild cognitive impairment. WMH=white matter hyperintensity

|  | **Patients in final experiment (N=813)** | **Patients with SCI (N=192; 23.6%))** | **Patients with MCI (N=207; 25.5%)** | **Patients with dementia (N=414; 50.9%)** |
| --- | --- | --- | --- | --- |
| Age in years, mean (SD) | 67.6 (8.5) | 63.0 (7.6) | 68.7 (8.81) | 69.1 (7.95) |
| Female, N (%) | 375 (46.1%) | 92 (47.9%) | 89 (43.0%) | 194 (46.9%) |
| Level of education (Verhage scale), median (IQR) (N=808) | 5 (4 – 6) | 5 (4 – 6) | 5 (4 – 6) | 5 (4 – 6) |
| Attention and executive functioning Z-scores, mean (SD) | -1.56 (0.7) | -1.04 (0.64) | -1.30 (0.62) | -1.94 (0.63) |
| Fazekas scale ≥ 2 | 370 (45.5%) | 63 (32.8%) | 101 (48.8%) | 206 (50.6%) |
| ≥ 1 Lacunar infarct(s) | 174 (21.4%) | 30 (15.6%) | 60 (29%) | 84 (20.3) |
| ≥ 1 Non-lacunar infarct(s) | 86 (10.6%) | 14 (7.3%) | 26 (12.6%) | 46 (11.1%) |
| ≥ 1 Microbleed(s) (N=807) | 356 (43.8%) | 73 (38%) | 93 (44.9%) | 190 (45.9%) |
| ≥ 1 Intracerebral hemorrhage(s) | 16 (2%) | 5 (2.6%) | 8 (3.9) | 3 (0.7%) |

[1] F. Verhage, “Intelligentie en leeftijd: Onderzoek bij Nederlanders van twaalf tot zevenenzeventig jaar,” 1964, [Online]. Available: https://library.wur.nl/WebQuery/titel/474508

Table S-2. Percentage abnormal neuropsychological test results
Test results are considered abnormal when Z-scores are below the 5th percentile, i.e. Z-scores < -1.65.

| **Neuropsychological test** | **Total, N** | **Abnormal, %** |
| --- | --- | --- |
| TMT-B | 602 | 41.2 |
| Digit Span Forward | 774 | 48.1 |
| Digit Span Backward | 768 | 91.0 |
| Phonemic fluency – NA^1^ | 63 | 27.6 |
| Phonemic fluency – DAT^2^ | 593 | 29.3 |
| Semantic fluency – Animals | 789 | 42.9 |

# Section B. Supplementary Tables and Figures


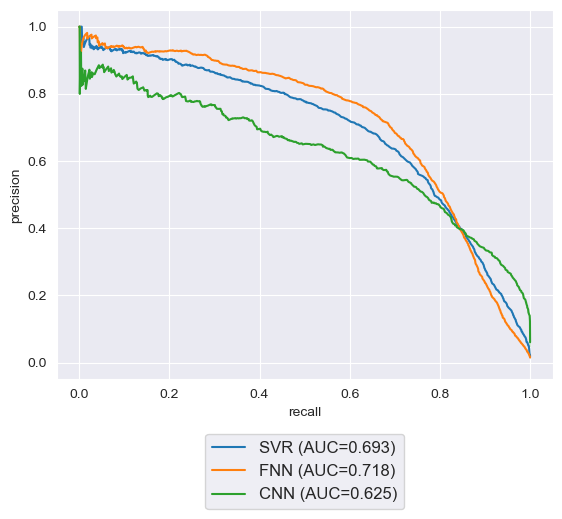


**Figure S-1. Basic cognitive simulations: precision-recall curves** Precision and recall were determined on the group-level attribution maps for each method. Precision-recall areas under the curve (AUC) for the group-level attribution maps are noted in the figure legend. The precision-recall curves for the SVR and FNN follow a similar pattern, while the curve for the CNN is noticeably different. The curves show that the CNN has more false positive values (suprathreshold values outside of the predefined regions of interest) at high thresholds compared to the SVR and FNN, resulting in a lower precision. An opposite effect can be observed at lower thresholds, where the SVR and FNN likely have more false positives than the CNN. the SVR=support vector regression. FNN=fully connected network. CNN=convolutional neural network.


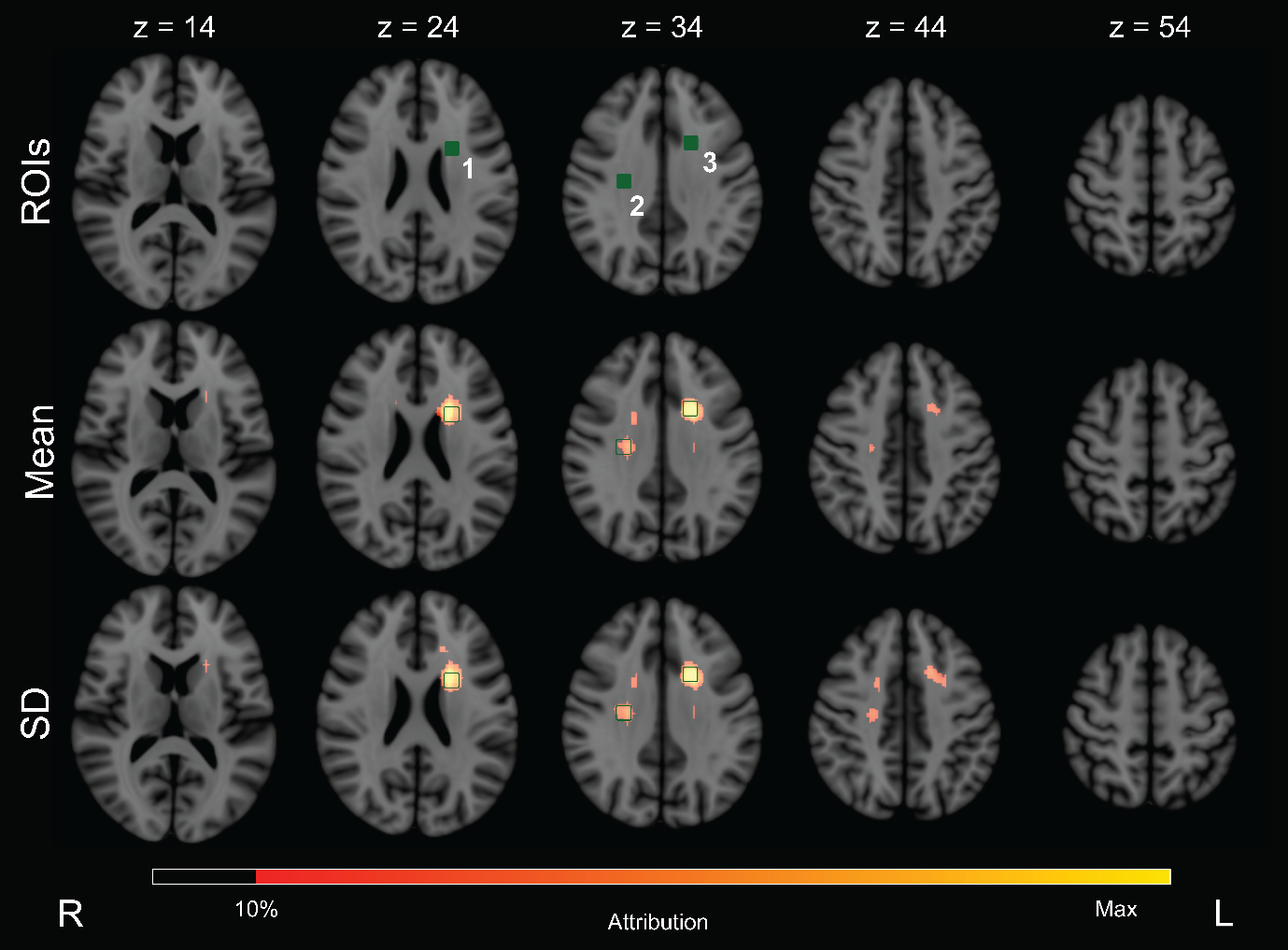


**Figure S-2.** **Basic cognitive simulations: mean and standard deviation results for the CNN** ROIs were the ground truth locations for the cognitive simulations. Equal weight was assigned to the three ROIs. The mean and standard deviation of individual attribution values are visualized on five axial slices of MNI-152 space, indicated by z. Mean and standard deviation maps look similar, both with the highest attribution values inside the three ROIs. SD=standard deviation.


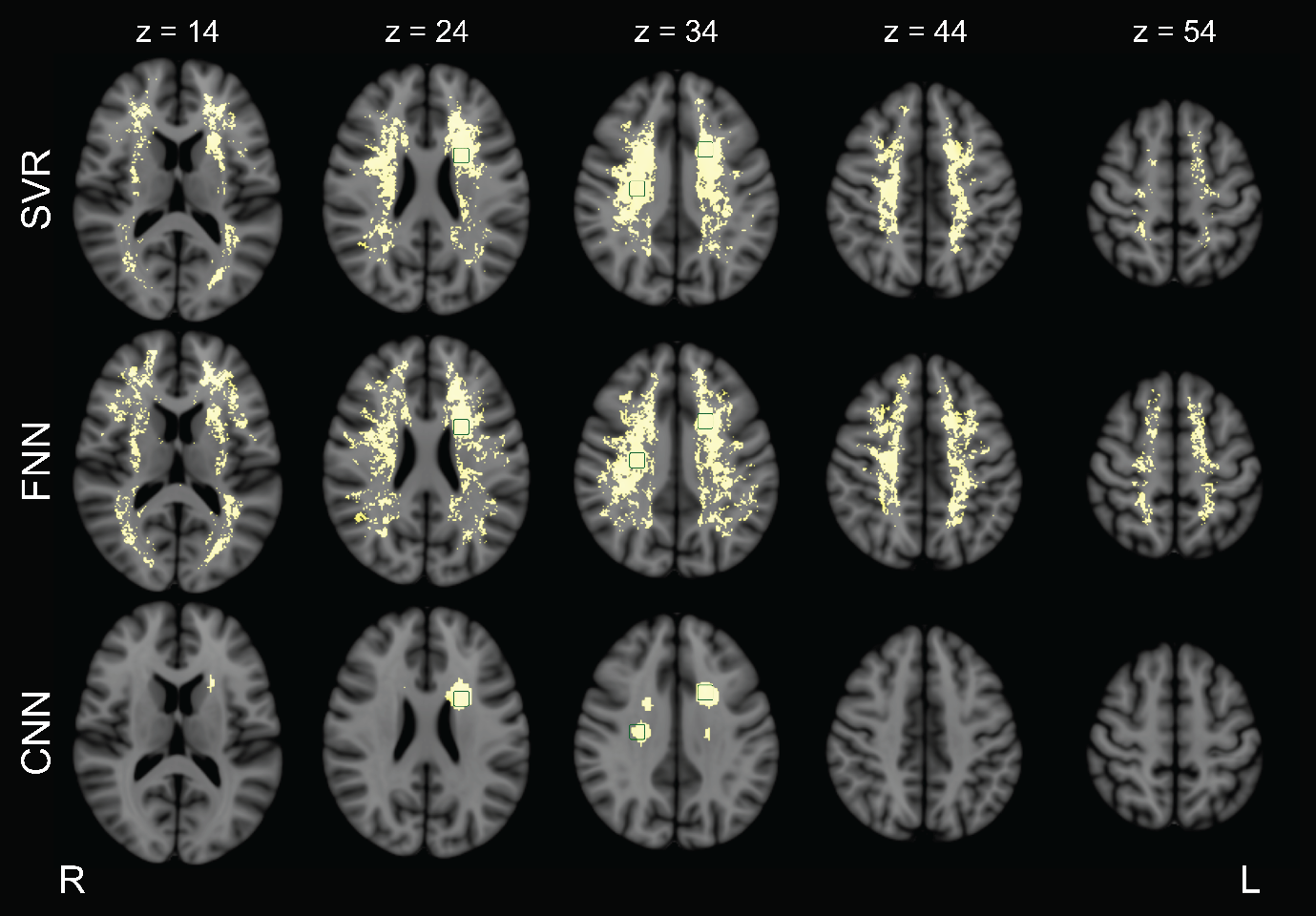


**Figure S-3.** **Basic cognitive simulations: group-level attribution maps with threshold at 10% of the maximum value**Z indicates the axial slices in MNI-152 space. SVR=support vector regression. FNN=fully connected network. CNN=convolutional neural network.


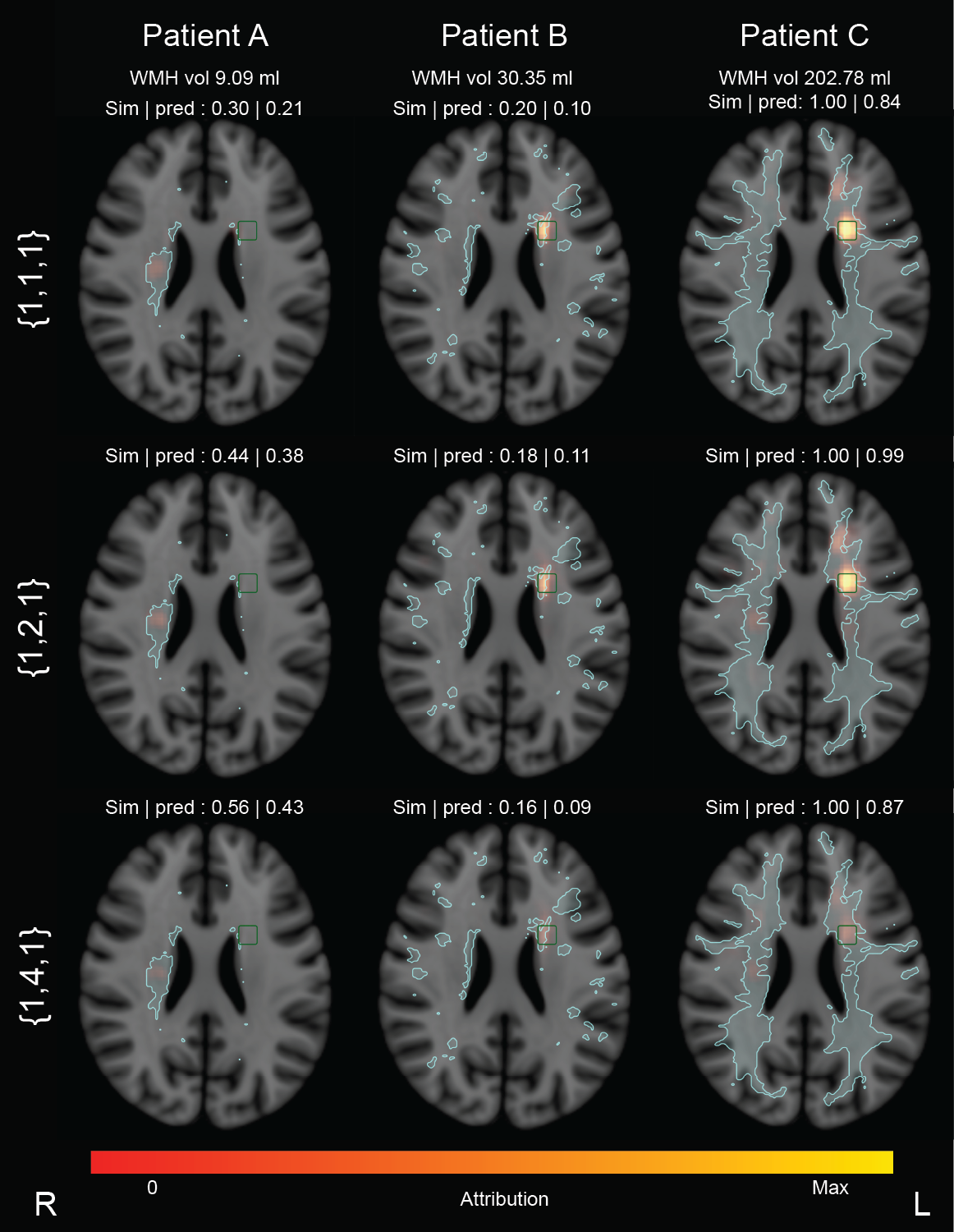


**Figure S-4. Basic cognitive simulations and simulation with modified regional weights: individualized lesion-symptom mapping results** Examples of individual attribution maps for three simulated cognitive scores projected on axial slice 24 of the MNI-152 template. WMH volume, simulated cognitive scores and predicted scores for each patient are denoted in the image. Simulated cognitive scores were based on WMH lesion load within the three ROIs. All scores are valued between 0 and 1, where 1 relates to a higher lesion load in the ROIs. WMH lesion contours are displayed in light blue, the predefined ROI contours in green. In this figure, only ROI 1 is visible. TOP ROW: For the basic cognitive simulations, lesion load in all three ROIs was weighted equally: {1,1,1}. It can be seen that the CNN with XAI correctly highlights the ROIs that contain lesions and no attributions are present in locations where there are no lesions. MIDDLE AND BOTTOM ROWS: Here, lesion load in ROI 2 was weighted more heavily than the other ROIs, depicted in row 2 {1,2,1} and 3 {1,4,1}. This yielded different simulated cognitive scores. Patient A has barely any WMH located in ROI 1 and shows a minimal amount of attribution in a WMH patch on the right side of the brain only. This is close to the location of ROI 2. Patient B does have some WMH located in ROI 1. Patient C has the highest WMH volume in this figure, the lesion covers a substantial portion of the brain and overlaps with ROIs almost completely, resulting in a score of 1.00 regardless of ROI weights. The intensity of the attribution values for patient B and C in ROI 1 decline when the contribution weight of ROI 2 is increased. WMH vol=white matter hyperintensity volume. Sim=simulated cognitive score. Pred=predicted cognitive score.

**Table S-3. Predictive performance results for simulation with modified regional weights**Predictions (N=821) made in the cross-validation were concatenated and evaluated against the artificial cognitive scores using an R^2^ obtained through an ordinary least squares. Similar to the results shown for experiment 1 based on basic cognitive simulations, predictive performance was consistently highest for the CNN. The SVR and FNN obtained similar predictive performance to one another. SVR=support vector regression. FNN=fully connected network. CNN=convolutional neural network.

| **ROI weights** | **{1,1,1}** | **{1,2,1}** | **{1,4,1}** |
| --- | --- | --- | --- |
| SVR | 0.867 | 0.862 | 0.839 |
| FNN | 0.856 | 0.851 | 0.831 |
| CNN | 0.951 | 0.942 | 0.943 |

**Table S-4. Predictive performance results for simulation with added noise**Predictions (N=821) made in the cross-validation were concatenated and evaluated against the artificial cognitive scores using an R^2^ obtained through an ordinary least squares. For all methods, predictive performance declined similarly with an increased noise fraction. SVR=support vector regression. FNN=fully connected network. CNN=convolutional neural network.

| **Noise fraction** | **0** | **0.25** | **0.50** | **0.75** |
| --- | --- | --- | --- | --- |
| SVR | 0.867 | 0.784 | 0.466 | 0.061 |
| FNN | 0.856 | 0.768 | 0.398 | 0.003 |
| CNN | 0.951 | 0.835 | 0.453 | 0.061 |

**Table S-5. Attribution map quality results for simulation with added noise**Precision-recall areas under the curve (AUC) were computed based on the (positive) group-level attribution maps for each method. Similar to the predictive performance, precision-recall AUC declined for all methods with an increased noise fraction. SVR=support vector regression. FNN=fully connected network. CNN=convolutional neural network.

| **Noise fraction** | **0** | **0.25** | **0.50** | **0.75** |
| --- | --- | --- | --- | --- |
| SVR | 0.693 | 0.629 | 0.111 | 0.027 |
| FNN | 0.718 | 0.510 | 0.050 | 0.024 |
| CNN | 0.625 | 0.544 | 0.154 | 0.025 |

# Section C. Additional analyses

## Dice similarity coefficient

The Dice similarity coefficient was computed between the ground truth ROIs and the attribution maps of each approach (in various experiments). This was done by applying a threshold at 50% of the maximum attribution value in the attribution maps, see Figure S-4.

Table S-6 shows that the CNN achieves a higher Dice similarity coefficient than SVR and the FNN. This is consistent in experiments that assign different weights to the ROIs or different noise fractions.

**Table S-6. Dice similarity coefficient at threshold of 50% of maximum attribution value**A threshold at 50% of the maximum attribution map was applied to all attribution maps. The Dice similarity coefficient was determined between the binary attribution maps and the ground truth ROIs. Dice similarity coefficients were highest for the CNN across all experiments, followed by SVR and the FNN. SVR=support vector regression. FNN=fully connected network. CNN=convolutional neural network.

| **Experiment** | **SVR** | **FNN** | **CNN** |
| --- | --- | --- | --- |
| 1. Basic | 0.367 | 0.252 | 0.508 |
| 2. ROI weights {1,2,1} | 0.326 | 0.126 | 0.413 |
| 2. ROI weights {1,4,1} | 0.267 | 0.153 | 0.359 |
| 3. Noise fraction 0.25 | 0.231 | 0.160 | 0.483 |
| 3. Noise fraction 0.50 | 0.018 | 0.014 | 0.069 |
| 3. Noise fraction 0.75 | 0.000 | 0.000 | 0.000 |

## False negatives

False negatives were determined in group-level attribution maps by counting the ROIs (N=3) without attribution values above 10% of the maximum attribution value (see Figure S-2). Results are provided in the Table S-7.

In most experiments, no false negative ROIs were found. SVR and the CNN did have false negatives in the experiment with the highest noise fraction. This is in line with the generally poor performance achieved for that particular experiment.

False negatives were also determined across individual patients (N=821). This was only possible for the CNN. Like in the group-level analysis, only attributions with values above 10% of the maximum for each patient’s map were taken into consideration. ROIs that did not contain WMH (N=1034 ROIs across all patients) were excluded. Results can be found in the Table S-8.

The number of true positive ROIs outweigh the number of false negative ROIs across all experiments.s False negatives increased with more noise and more false negatives were detected in the experiment with modified ROI weight in which the contribution of ROI 2 was weighted four times more than the other ROIs. When the contribution of ROI 2 was weighted twice as much as other ROIs, the number of false negatives actually decreased. This is also in line with the group attribution maps seen in the main manuscript.

**Table S-7. False negative ROIs**False negatives were determined in group-level attribution maps by counting the ROIs (N=3) without attribution values above 10% of the maximum attribution value. In most experiments, no false negative ROIs were found. SVR and the CNN did have false negatives in the experiment with the highest noise fraction. This is in line with the generally poor performance achieved for that particular experiment. SVR=support vector regression. FNN=fully connected network. CNN=convolutional neural network.

| **Experiment** | **SVR** | **FNN** | **CNN** |
| --- | --- | --- | --- |
| 1. Basic | 0 | 0 | 0 |
| 2. ROI weights {1,2,1} | 0 | 0 | 0 |
| 2. ROI weights {1,4,1} | 0 | 0 | 0 |
| 3. Noise fraction 0.25 | 0 | 0 | 0 |
| 3. Noise fraction 0.50 | 0 | 0 | 0 |
| 3. Noise fraction 0.75 | 3 | 0 | 2 |

**Table S-8. False negative ROIs for individual patients**False negatives were determined in individual-level attribution maps by counting the ROIs (N=3) without attribution values above 10% of the maximum attribution value. ROIs that did not contain WMH (N=1034 ROIs across all patients) were excluded. The number of true positive ROIs outweigh the number of false negative ROIs. False negatives increase with higher noise levels. SVR=support vector regression. FNN=fully connected network. CNN=convolutional neural network.

| **Experiment** | **False negative ROIs** | **True positive ROIs** |
| --- | --- | --- |
| 1. Basic | 89 | 1340 |
| 2. ROI weights {1,2,1} | 69 | 1360 |
| 2. ROI weights {1,4,1} | 116 | 1313 |
| 3. Noise fraction 0.25 | 94 | 1335 |
| 3. Noise fraction 0.50 | 107 | 1322 |
| 3. Noise fraction 0.75 | 416 | 1013 |


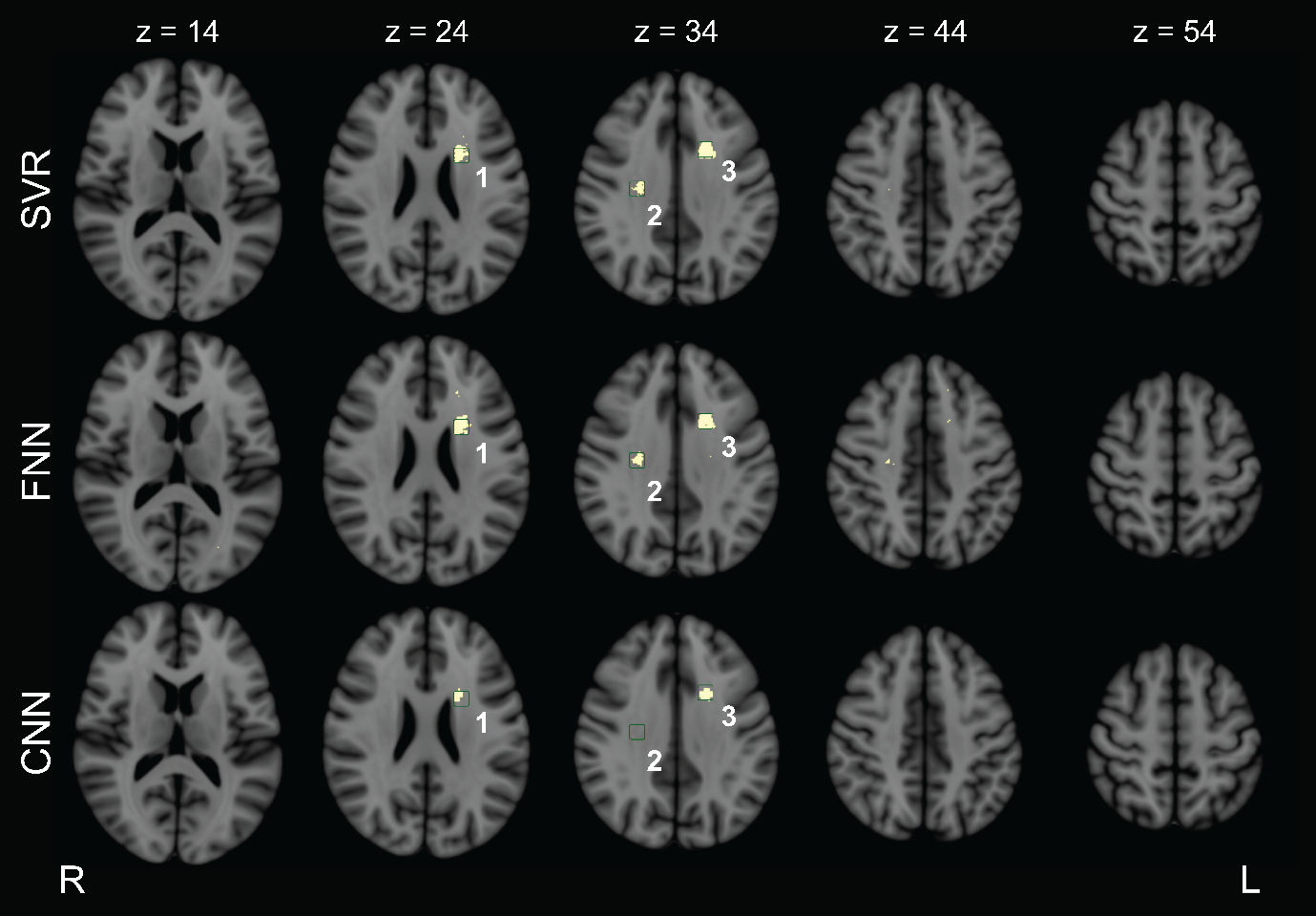


**Figure S-5.** **Basic cognitive simulations: group-level attribution maps with threshold at 50% of the maximum value**
Z indicates the axial slices in MNI-152 space. SVR=support vector regression. FNN=fully connected network. CNN=convolutional neural network.

## White matter tracts with the highest attribution values

The primary objective of this study was to evaluate the feasibility of using a CNN with XAI in a simulation study. The study was not designed to provide definite answers on the regions involved.

Table S-9 provides an overview of the top three white matter tracts with the highest attribution values relative to the tract volume, based on the Johns Hopkins University ICBM diffusion tensor imaging atlas. The top three white matter tracts are consistent across the three methods, although the order of appearance differs.

**Table S-9. Top three white matter tracts with highest attribution values relative to tract volume**
The Johns Hopkins University ICBM diffusion tensor imaging atlas was to identify the tracts. The top three white matter tracts are consistent across the three methods, although the order of appearance differs.s

| SVR | FNN | CNN |
| --- | --- | --- |
| 1. Right inferior fronto-occipital fasciculus 2. Forceps major 3. Left inferior fronto-occipital fasciculus | 1. Left inferior fronto-occipital fasciculus 2. Forceps major 3. Right inferior fronto-occipital fasciculus | 1. Forceps major 2. Left inferior fronto-occipital fasciculus 3. Right inferior fronto-occipital fasciculus |

# Section D. Additional experiments with simulated cognitive data

To test whether results remain consistent when considering ROIs with low lesion prevalence, two additional experiments were performed:

1. Six new ROIs with a larger variety in mean WMH prevalence, ranging from 0.036 to 0.184; and
2. Three ROIs with a low WMH prevalence, ranging from 0.039 - 0.065.

The mean WMH prevalence for each of the ROIs are provided in the tables below.

|  | Mean WMH prevalence |  |  | Mean WMH prevalence |
| --- | --- | --- | --- | --- |
|  | 1. Six ROIs |  |  | 2. Three ROIs |
| ROI A | 0.065 |  | ROI A | 0.065 |
| ROI B | 0.077 |  | ROI C | 0.039 |
| ROI C | 0.039 |  | ROI D | 0.041 |
| ROI D | 0.041 |  |  |  |
| ROI E | 0.188 |  |  |  |
| ROI F | 0.159 |  |  |  |

Simulated cognitive scores were determined by the sum of the WMH lesion load inside the ROIs and a noise fraction of 0.25, using the same method as described in section 2.4.3. ‘Experiment 3: Simulation with implemented noise’ of the manuscript.
Noise was included because predictive performance in experiments without noise is high for all methods, which could make differences in performance more difficult to detect because of a potential ceiling effect.

Predictive performance for SVR, the FNN and CNN was similar for the experiment with six ROIs (R^2^ > 0.8), see table below. SVR and the FNN achieved a slightly higher predictive performance than that was achieved with the original ROIs in the manuscript. In the second experiment with three ROIs of lower WMH prevalence, the CNN’s predictive performance was noticeably higher than the benchmarks. Compared to the performance with the original ROIs in the manuscript, SVR and the FNN especially obtained a lower predictive performance.

| Predictive performance in R^2^ | | |
| --- | --- | --- |
|  | 1. Six ROIs 25% noise | 2. Three ROIs 25% noise |
| SVR | 0.818 (0.036) | 0.693 (0.071) |
| FNN | 0.808 (0.039) | 0.685 (0.079) |
| CNN | 0.838 (0.034) | 0.806 (0.075) |

As can be seen in the table below, SVR achieved higher precision-recall area under the curve (AUC) than the FNN and CNN for both experiments. Precision-recall AUC values for the experiment with six ROIs was considerably lower than for the experiment with three ROIs. Results for the experiment with three ROIs aligns with results achieved for the original ROIs and 25% noise in the original manuscript.

| Precision-recall area under the curve | | |
| --- | --- | --- |
|  | 1. Six ROIs 25% noise | 2. Three ROIs 25% noise |
| SVR | 0.316 | 0.687 |
| FNN | 0.227 | 0.545 |
| CNN | 0.265 | 0.501 |

Figures S-5 and S-6 present the group-level attribution maps for both experiments. In the experiment with six ROIs (Figure S-5), all models seem to focus most on ROIs B and E, which both have high WMH prevalence. ROI A, D, and F show limited attribution in all methods, which is confirmed by the relative ROI attributions in the table below. This is not consistent with the average WMH prevalence in these ROIs, e.g. ROI F has a relatively high WMH prevalence. But what can be observed is that ROIs A and F are more distant from the other ROIs. Relative attribution for ROI C is lower in the CNN than for the benchmarks, while attribution values are relatively high in ROI E, which does coincide with a lower lesion prevalence.

| 1. Relative attribution values in ROIs | | | |
| --- | --- | --- | --- |
|  | SVR | FNN | CNN |
| ROI A | 0.043 | 0.050 | 0.028 |
| ROI B | 0.255 | 0.300 | 0.257 |
| ROI C | 0.189 | 0.232 | 0.079 |
| ROI D | 0.056 | 0.063 | 0.013 |
| ROI E | 0.400 | 0.309 | 0.608 |
| ROI F | 0.059 | 0.047 | 0.015 |

Figure S-6 shows that all methods are able to highlight the three ROIs with low WMH prevalence. The table with relative attribution values in ROIs below, shows that the CNN with XAI assigned higher attribution values in the ROIs with higher WMH prevalence, while SVR and the FNN show similar attribution values for all ROIs regardless of the difference in WMH prevalence.

| 2. Relative attribution values in ROIs | | | |
| --- | --- | --- | --- |
|  | SVR | FNN | CNN |
| ROI A | 0.323 | 0.342 | 0.518 |
| ROI C | 0.329 | 0.332 | 0.263 |
| ROI D | 0.348 | 0.326 | 0.219 |

Overall, from these analyses we observe that the CNN is more sensitive to lesion prevalence than SVR and the FNN.


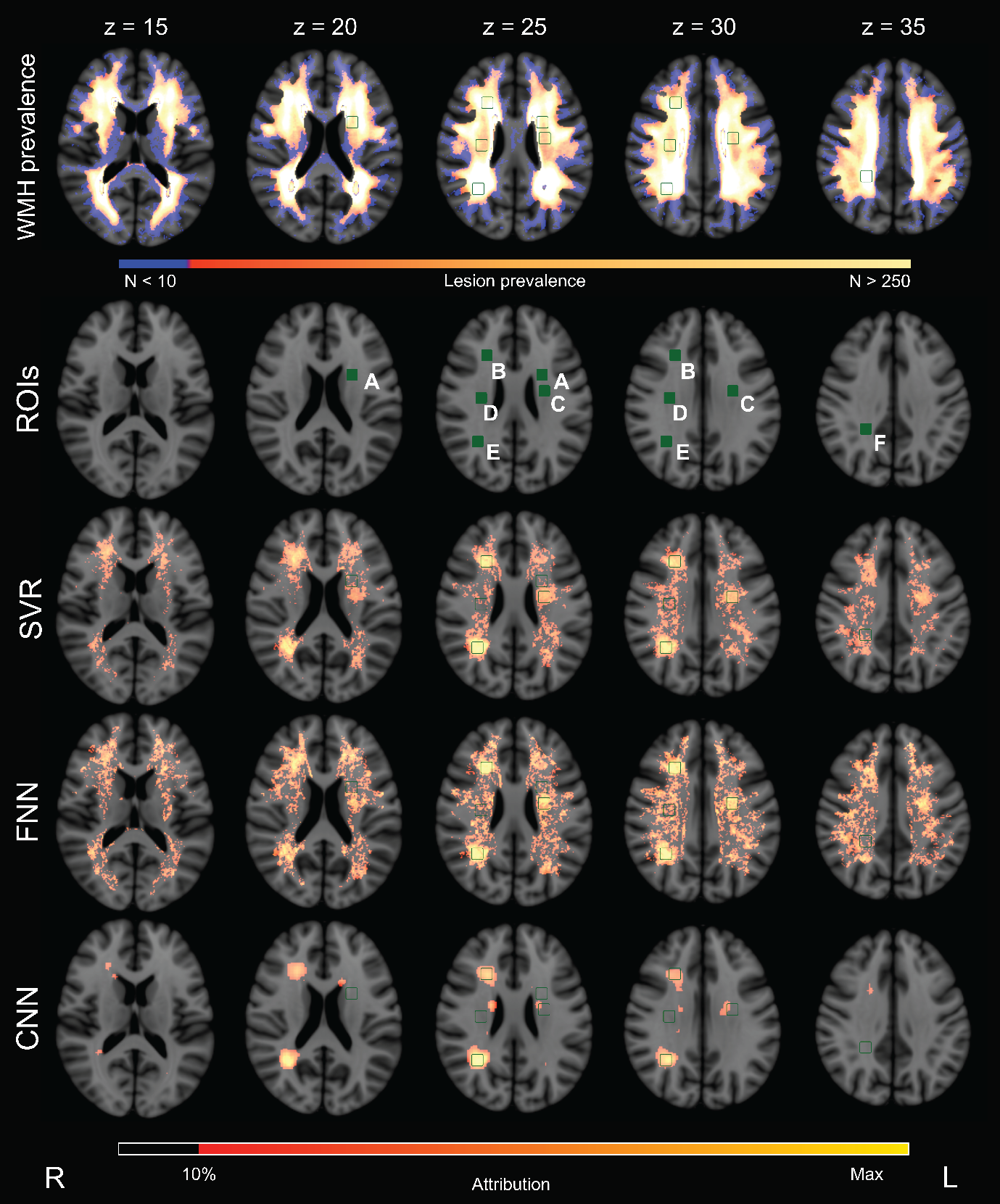


**Figure S-6.** **Six ROIs with varying WMH prevalence and added noise: group-level lesion-symptom mapping results**
Positive group-level attribution values are visualized on five axial slices of MNI-152 space. Axial slices are indicated by z. The centres of the ROIs A-F were on axial slice 24, 27, 28, 30, 26, and 36, respectively. All three methods assigned the highest attribution values to ROIs B and E. ROIs A, D, and F on the other hand, received limited attribution values. Apart from this, attribution values appear to be more widely distributed for SVR and the FNN compared to the CNN. WMH=white matter hyperintensities. ROIs=regions of interest. SVR=support vector regression. FNN=fully connected network. CNN=convolutional neural network.


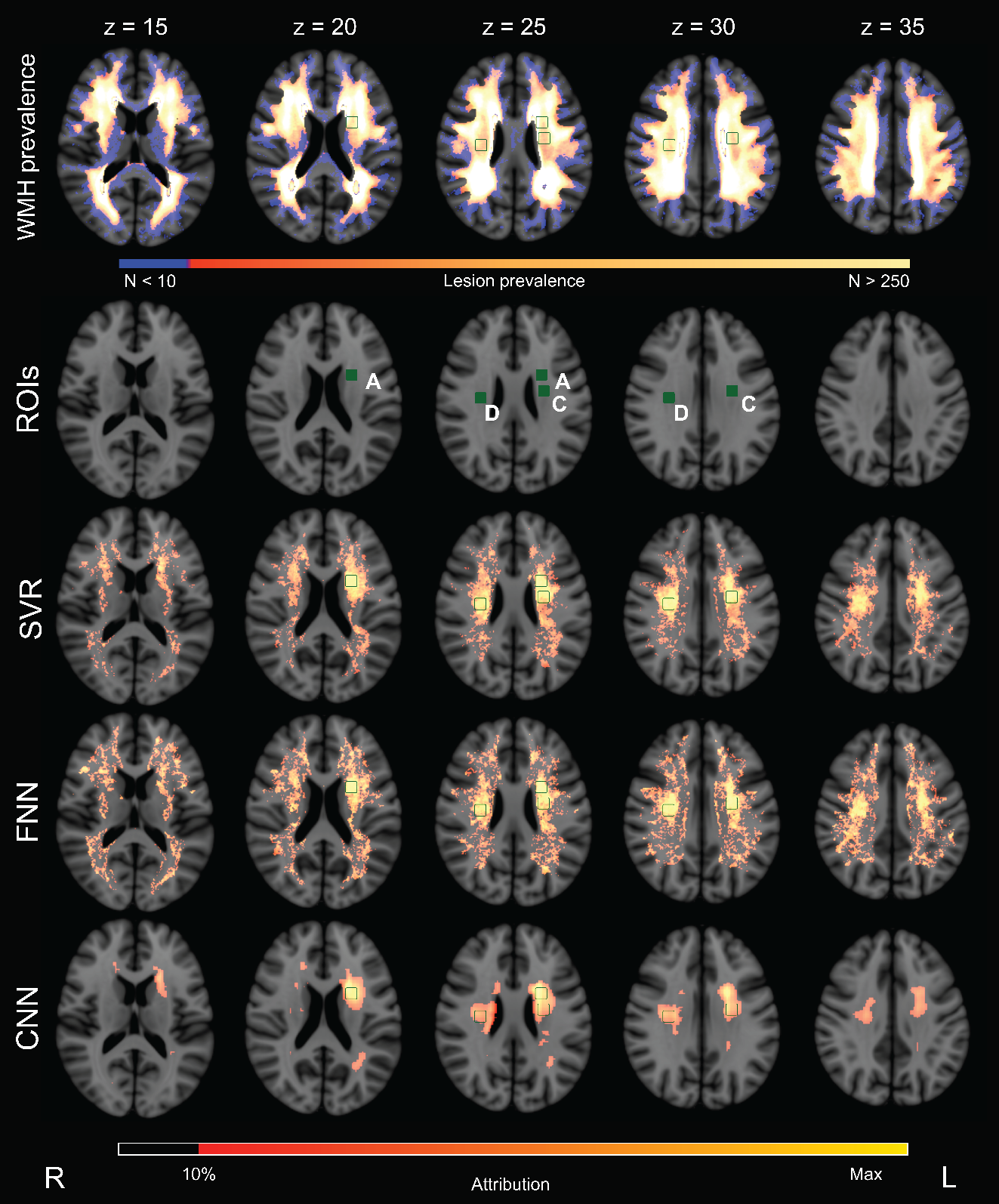


**Figure S-7.** **Three ROIs with low WMH prevalence and added noise: group-level lesion-symptom mapping results**
Positive group-level attribution values are visualized on five axial slices of MNI-152 space. Axial slices are indicated by z. The centres for ROI A, C, and D were on axial slice 24, 28, and 30, respectively. All three ROIs were highlighted by the methods. In the CNN however, it is clear that attribution values for ROI A were higher than for the other two ROIs. This coincides with a higher mean WMH prevalence in ROI A. Attribution values for SVR and the FNN appear similar for all ROIs. Next to that, attribution values appear to be more concentrated on the ROIs in the CNN. False positive attributions are more widely distributed for SVR and the FNN. WMH=white matter hyperintensities. ROIs=regions of interest. SVR=support vector regression. FNN=fully connected network. CNN=convolutional neural network.

# Section E. Additional analysis on neuropsychological tests

Lesion-symptom mapping was performed for two separate neuropsychological tests that contribute to the attention and executive functioning domain Z-scores: trail making test part B and semantic fluency. The same methods were used as for the attention and executive functioning domain as described in the main manuscript.

Results show that predictive performance of SVR was best in the trail making test part B. See Table S-9 for an overview of predictive performance for both neuropsychological tests. For semantic fluency, predictive performance of SVR and the CNN were comparable, while the FNN performed worse. This is in line with the results obtained on the attention and executive functioning domain. Figures S-7 and S-8 show the group-level attribution maps obtained using the three methods for the trail making test part B and semantic fluency. Visually, similar locations are highlighted for both neuropsychological tests, which seem to overlap with the locations that were marked for the attention and executive functioning domain.

**Table S-9. Predictive performance results for trail making test part B and semantic fluency** Predictions (N=813) made in the cross-validation were concatenated and evaluated against the normalized scores using an R^2^ obtained through an ordinary least squares. SVR=support vector regression. FNN=fully connected network. CNN=convolutional neural network.

|  | Trail making test part B | Semantic fluency |
| --- | --- | --- |
| SVR | 0.210 (0.039) | 0.057 (0.023) |
| FNN | 0.006 (0.008) | 0.010 (0.007) |
| CNN | 0.153 (0.026) | 0.059 (0.019) |


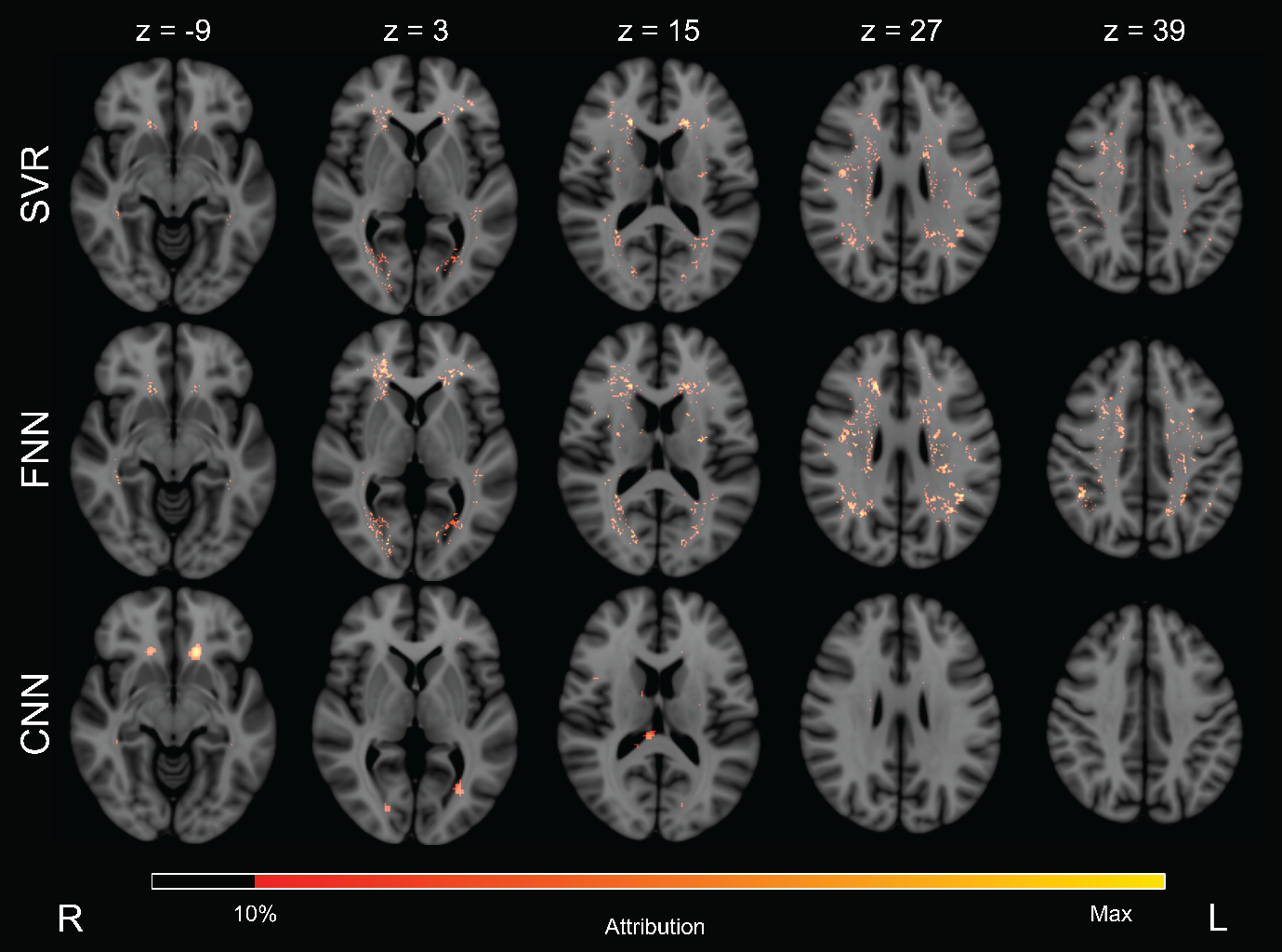


**Figure S-8.** **Trail making test part B: group-level lesion-symptom mapping results** Positive group-level attribution maps visualized on five axial slices of MNI-152 space for all three models. Axial slices are indicated by z. Since there is no ground truth on the actual associations with the real cognitive scores, this is a qualitative assessment. SVR=support vector regression. FNN=fully connected network. CNN= convolutional neural network.


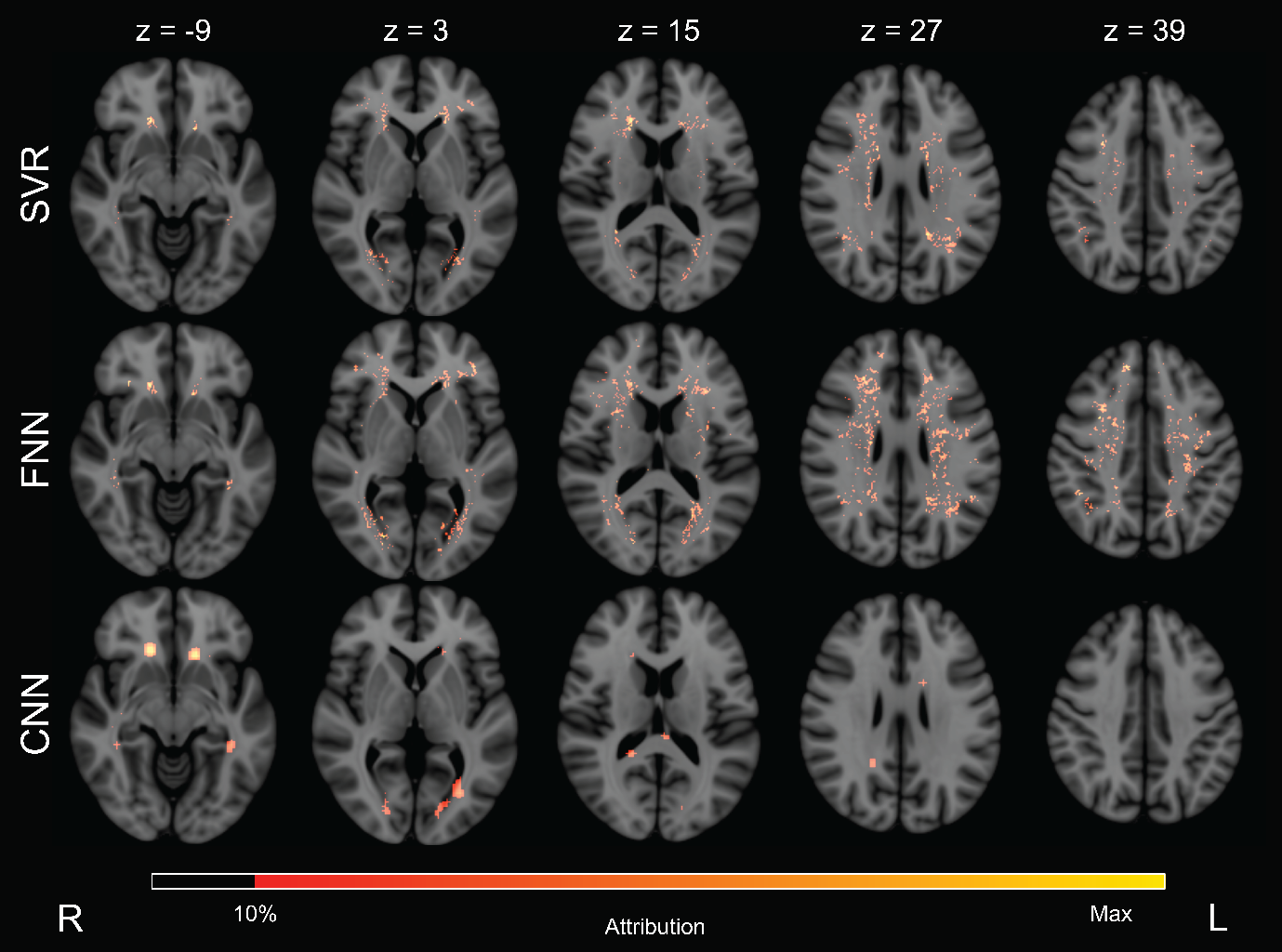


**Figure S-9. Semantic fluency: group-level lesion-symptom mapping results** Positive group-level attribution maps visualized on five axial slices of MNI-152 space for all three models. Axial slices are indicated by z. Since there is no ground truth on the actual associations with the real cognitive scores, this is a qualitative assessment. SVR=support vector regression. FNN=fully connected network. CNN= convolutional neural network.
